# Psychological determinants of GenAI adoption for foreign language education: an extended UTAUT2 model and sentiment analysis approach

**DOI:** 10.3389/fpsyg.2025.1622926

**Published:** 2026-01-09

**Authors:** Huan Wang, Tian Liu

**Affiliations:** Foreign Studies College, Northeastern University, Shenyang, China

**Keywords:** GenAI adoption, foreign language education, UTAUT2, AI anxiety, sentiment analysis, technology adoption, psychological determinants

## Abstract

**Introduction:**

The use of Generative Artificial Intelligence (GenAI) in foreign language education is also a paradigm shift; however, psychological factors that affect its adoption are not well understood. While established models such as the Unified Theory of Acceptance and Use of Technology 2 (UTAUT2) describe technology adoption in general, they often fail to capture the unique emotional and cognitive reactions triggered by AI.

**Methods:**

This paper thus presents an important construct to explain the special emotional and cognitive reactions triggered by the learning of human-AI collaboration: AI anxiety. Structural equation modeling (SEM) was used to analyze survey data (*N* = 632), and sentiment analysis was used for online reviews.

**Results:**

The results indicate that Performance Expectancy, Effort Expectancy, and Social Influence positively affect Behavioral Intention. On the other hand, AI Anxiety and Habit significantly negatively affect Behavioral Intention. Sentiment analysis supported these findings, demonstrating positive public sentiment and anxiety and complexity associated with adopting decision-making.

**Discussion:**

The research shows that integrating GenAI into learning is not a functional interaction but a complex psychological interaction. The implications of this research are important for people developing technologically sophisticated yet psychologically attuned AI tools.

## Introduction

**Figure 1:**
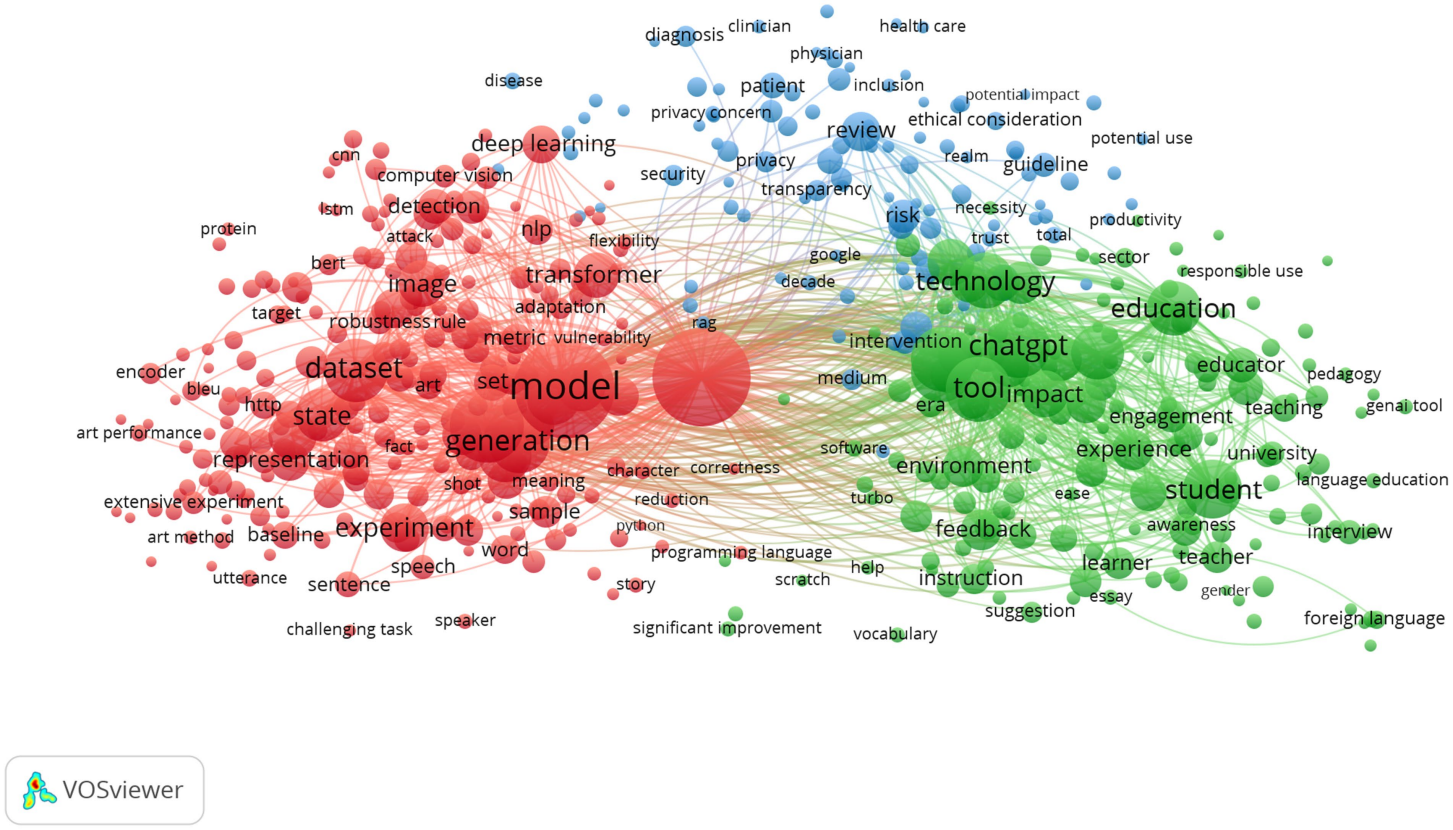
Keywords with the greatest total link strength.

What if your personal tutor was available around the clock, could create a conversation on any subject you could think of, and never lost her or his temper? This is no longer science fiction fantasy, but the reality of GenAI in foreign language education. AI tutoring, such as DeepSeek, is a fast-emerging reality in global education (Guo et al., 2025; Liu et al., 2024). It is argued, for instance, that how a system presents itself, mimics (real-time feedback) and engages (affect) make personalization possible, which can compensate for the difficulties that arise from human teaching (lack of proper pronunciation, engagement, motivation, etc.). The revolution can be quantified as a recent search in the Web of Science Core Collection, which returns 1,538 scholarly records containing the string “GenAI language learning,” which shows an exponentially increased attention in the literature to this topic. As shown in Figure 1, created using VOSviewer, bibliometric mapping reveals three major research clusters, model development, social applications, and educational integration, in which the most common co-occurring keywords relate to student engagement and teacher adoption. The evidence is clear: GenAI is no longer an add-on classroom tool-it is redefining the very concept of what it means to teach and learn a language.

Despite increasing interest, there is a lack of in-depth research on the motivational and psychological barriers that ultimately govern the acceptance of GenAI in foreign language education. As good as UTAUT2 is for technology adoption in general, the traditional model struggles to conceptualize the role of these psychological dynamics in educators’ and learners’ decision-making processes regarding acceptance or rejection of an AI collaborator. This paper proposes an expanded UTAUT2 model that incorporates the important dimension of AI anxiety to address this gap. By assessing users’ behavioral intentions and affective responses, as well as the perceived alignment of AI competencies and human-centered classroom responsibilities, this study provides a novel insight into the psychological transaction that is the central mechanism of human–AI collaboration in education. The following two fundamental questions guide the research:

RQ1. What psychological factors drive or hinder the adoption of GenAI in foreign language education?

RQ2. How does the public perceive GenAI tools like DeepSeek?

### Theoretical framework

The present research will be based on an extended UTAUT2 model, in which AI anxiety is treated as the simplest psychological factor. While UTAUT2 traditionally emphasizes behavioral predictors of technology use (Venkatesh et al., 2012), we redefine its constructs in terms of psychological theories to better capture the cognitive, motivational, and emotional processes underlying GenAI acceptance. The fact that AI anxiety is treated as an independent psychological concept reflects an emotional attachment to technology adoption, i.e., the fear, uneasiness, and techno-phobia that may be present among teachers and students when interacting with more autonomous systems. There are diverse causes of this fear: fear of technological substitution, algorithmic opacity, and a sense of threat to professional identity. The suggested theoretical integration provides a more detailed explanation of the communication between cognitive assessment and emotional responses to ascertain behavioral intentions to use GenAI in the teaching of foreign languages, thereby bridging a gap in core psychological interactions between humans and artificial intelligence in the learning process.

### Research model and hypotheses

#### AI anxiety (AA)

The conceptualization of AI Anxiety as a feeling of fear or apprehension about the use of AI systems is a key determinant of technological adoption, especially in the subtle sphere of foreign language learning (Heinssen et al., 1987; Johnson and Verdicchio, 2017). Based on the literature about computer anxiety, AA in this respect goes beyond the general technophobia into the realm of fears of excessive dependency, the concern about the message of the AI as accurate and culturally sensitive, as well as the feeling that the humanistic and interactive essence of the learning of the language is under threat. There is also substantial literature that views AA as an essential obstacle to the implementation of educational technologies, especially in the field of foreign languages (Abu-Rayyash, 2024; Saehu and Hkikmat, 2025). AA can be conceptualized as an emotional reaction to fear of AI systems, but it goes beyond general technophobia to encompass specific fears related to learning (Rathakrishnan et al., 2024; Zhai et al., 2024). They are the fear of over-reliance as an impediment to personal mastery, fears about the validity and cultural sensitivity of AI-generated content, and a sense of the danger to the humanistic, interactive essence of language acquisition (Zhi and Wang, 2024; Zhou and Hou, 2024). In this view, the overall finding is that an increase in AA is associated with decreases in Perceived Ease of Use and Usefulness, and thus a decrease in Behavioral Intention (BI) to use AI tools.

However, empirical evidence demonstrates that contradictory results indicate the complexity of AA. Against the perception of AA as unavoidably inhibitory, an alternative body of research proposes that an intermediate amount of AA is not only potentially helpful but will produce a state of productive struggle that will encourage learners to be more critical and reflective in their learning process, increasing their levels of engagement and metalinguistic awareness (Elmoon, 2025; Lachance and Honigsfeld, 2022). Another controversial issue is which users are most affected. Whereas there is a school of thought that AA is highest among low-proficiency learners who feel vulnerable to the technology (Qiao and Zhao, 2023), other studies have shown that advanced users have significantly higher levels of AA because of their increased capacity to detect the subtle mistakes of AI and other limits on ethics, which forms a U-shaped debate on the proficiency of the users (Frenkenberg and Hochman, 2025). Thus, the first significant gap is the need to replace AI Anxiety as a key construct with an extended UTAUT2 model to comprehensively describe user behavior regarding Generative AI in the international language learning context.

*H1*: AI Anxiety will exert a significant negative influence on BI when students use DeepSeek in foreign language education.

### Performance expectancy (PE) and effort expectancy (EE)

PE is the belief that technology applications enhance performance in academic or professional settings (Venkatesh et al., 2003). The educational motive for PE is a primary factor in determining how students and educators will adopt AI-powered instructional tools in their classrooms (Rahiman and Kodikal, 2024). AI adaptive learning systems, automated tutoring and assessment systems enhance learning outcomes and student engagement, and strengthen the impact of PE on BI (Miller et al., 2025; Shoaib et al., 2024). EE directly affects technology adoption, especially in digital education platforms that require intuitive interfaces and minimal mental effort to keep students engaged in the learning process (Al-kfairy et al., 2024). Students have shown greater intent to use educational technologies they see as embedded in their existing systems and easy to use, according to Ayanwale and Ndlovu (2024). PE and perceived usefulness have a positive predictive impact on acceptance. However, this relationship is influenced to varying degrees by factors such as users’ digital capabilities and experience with AI learning tools. This research builds on existing work by analyzing the relationships among PE and EE factors, the adoption of the DeepSeek model in education, and a current literature gap on AI use in personalized automatic learning environments.

*H2*: PE will positively and significantly predict BI when students use DeepSeek in foreign language education.

*H3*: EE will positively and significantly predict BI when students use DeepSeek in foreign language education.

### Social influence (SI) and facilitating conditions (FC)

Through the use of AI-based educational technologies, users experience behavioral change, which is dependent on two major factors: SI and FC. Influences on the adoption of educational tools are driven by the amount of support from key persons such as peers, educators, and institutions (Venkatesh et al., 2003). Peer and Institutional support in the adoption of AI-based tutoring systems: Chatterjee et al. (2024) and Farhi (2024) found results similar to those of previous research on peer and institutional support. SI’s impact on decision-making depends on the factors that dominate the procedure, such as autonomy and self-efficacy (Milicevic et al., 2024). FC, such as institutional and technical assistance, is important in the adoption of AI in education. Bhat et al. (2024) demonstrate that teachers using ChatGPT for curriculum development have improved learning outcomes, though this effect is not consistent without appropriate infrastructure, information technology support, and training. Many studies have shown that the FC indicator has excellent predictive ability in the implementation of adoption processes. The extent of this issue’s influence depends on users’ digital experience with such systems (Erdem and Çelik, 2024).

*H4*: SI will positively and significantly predict BI when students use DeepSeek in foreign language education.

*H5*: FC will positively and significantly predict UB when students use DeepSeek in foreign language education.

### Hedonic motivation (HM) and price value (PV)

Students adopt educational technology for hedonic reasons, as it provides them with internal pleasure (Kaczmarek, 2017). Through AI technology-based enjoyable experiences, student motivation increases, leading to their regular adoption of AI education systems (Chiu et al., 2024). Scientific evidence indicates that students continue to use educational technology when it is interesting to them (Abedi et al., 2024). Students evaluate the acceptance of AI-driven learning tools using PV, which represents their assessment of the benefits and costs of adopting the technology (Monroe, 2012). Student acceptance of AI-enhanced platforms in online education and adaptive learning systems significantly increaseswhen they are perceived as having high educational value at acceptable costs, according to studies by Abderrezzaq and Fatima (2024). The research examines the HM and PV factors to understand student acceptance of DeepSeek AI in educational settings.

*H6*: HM will positively and significantly predict BI when students use DeepSeek in foreign language education.

*H7*: PV will positively and significantly predict BI when students use DeepSeek in foreign language education.

#### Habit (HB)

Habit refers to behavioral patterns learned through automaticity, repetition, and repetition, which play an important role in the implementation of educational technology (Mixon, 1980). The study establishes a habit as an unconscious behavior when a user turns to AI-based learning machines like DeepSeek through an intuitive set of behaviors. Studies have demonstrated that habit plays a significant role in the adoption of technology, particularly in students’ use of e-learning programs and digital learning models. In an examination of the use of AI-powered tutoring systems, Han et al. (2024) showed that students who use such systems more organically are more likely to maintain their use, and the same was found by Maheshwari (2024) for mobile learning applications. Research shows that habits may encourage users to engage in activities. However, they may also hinder users’ adaptation to new system functions or new ways of learning within a short period of time (Sembiring et al., 2024). The overuse of AI educational tools diminishes students’ critical thinking and self-regulated learning skills, thereby impeding their ability to evaluate content (Yusuf et al., 2024). Resolving this AI learning paradox is very important to ensure that AI education systems deliver maximum value, as it affects whether repeating a way of doing something helps students learn better or hurts cognitive and learning development.

*H8*: Habits will negatively and significantly predict BI when students use DeepSeek in foreign language education.

*H9*: Habits will negatively and significantly predict UB when students use DeepSeek in foreign language education.

### Behavioral intention (BI)

**Figure 2:**
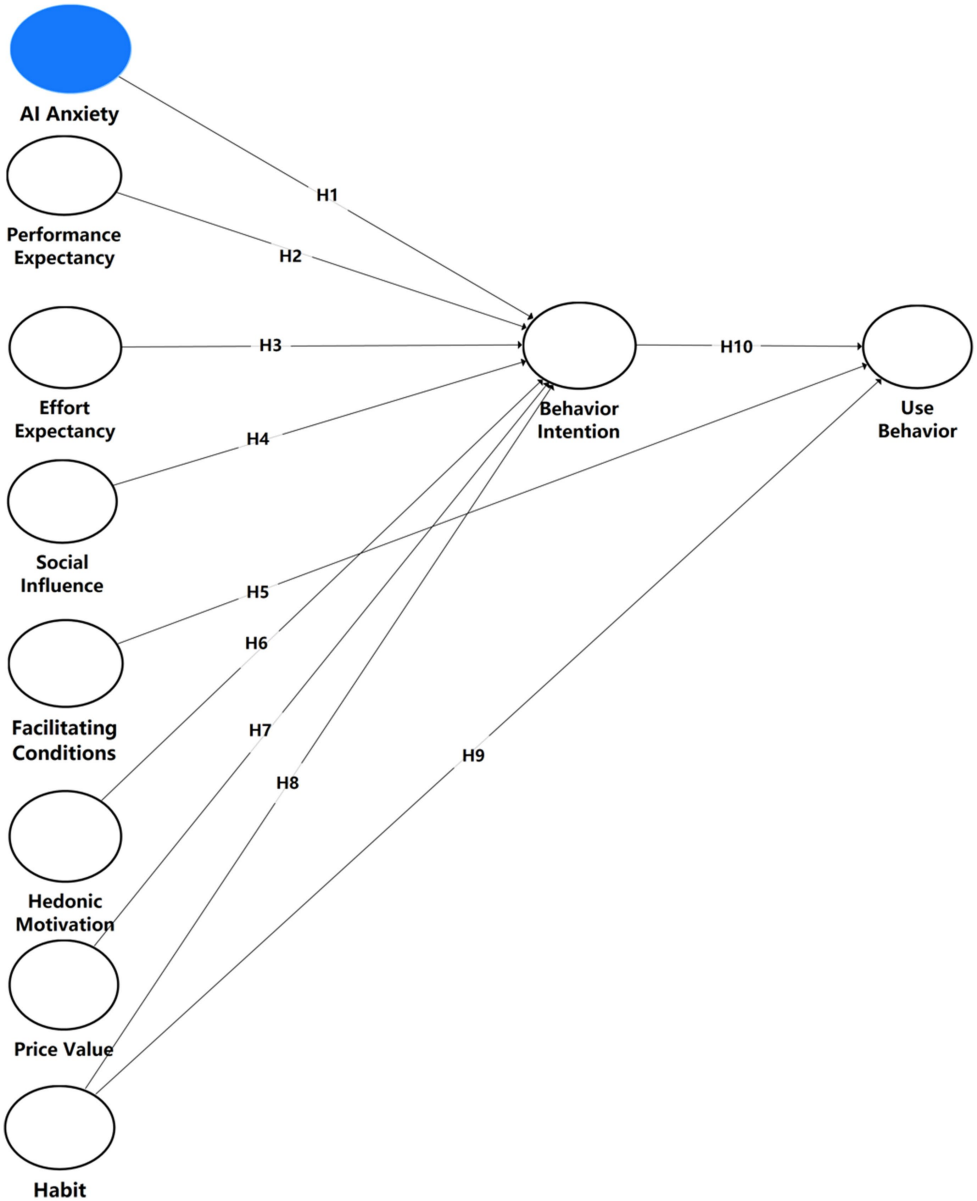
Proposed research model.

BI is the individual’s willingness to try a particular behavior (Venkatesh et al., 2003). BI is essential in determining students’ adoption decisions for AI-based educational systems. According to research papers, BI has a significant role in the technological adoption in education (Dangelico et al., 2024; Hemdan and Zhang, 2024). Different graduate students at different stages of their journey have varying levels of BI, as reflected in their views towards the EE and SI factors in digital learning environments (Tian et al., 2024). Research evidence proves that BI is a prediction tool for adopting educational technology. The effect of building individual technology competence on student retention for AI-based learning models in tailored, individualized educational contexts warrants further research (Sasidharan and Venkatakrishnan, 2024). AI deployment in education must address this research gap to ensure optimal implementation of AI systems in academic environments. The final research model is presented in Figure 2.

*H10*: BI will positively and significantly predict UB when students use DeepSeek in foreign language education.

### Sentiment analysis in educational technology

By combining PLS-SEM with sentiment analysis, the validity of the conclusions is further strengthened, revealing how users feel about the technology. Sentiment analysis has proven to be an important method for assessing public attitudes toward educational AI technologies, although people express both hopefulness and concerns about ethical dilemmas and technical confidence (Adeshola and Adepoju, 2024; Mahmoud, 2024). AI sentiment analysis tools help identify students’ preferred teaching methods, as they favor clear, interactive educational methods (Alvarez-alvarez and Falcon, 2023). A sentiment analysis of students’ online learning experiences reveals a mix of positive and negative feedback that acknowledges the flexibility of digital platforms yet emphasizes engagement difficulties and high student attrition (Chen et al., 2024; Saneinia et al., 2024). Therefore, this research also delved into the acceptance of the DeepSeek AI model in educational environments by analyzing public opinion and perceived benefits.

## Methodology

This research is designed as a mixed-methods research, involving survey data (based on SEM) and sentiment analysis of online discussions. The main application of this integration is triangulation: the survey captures causal relations and users’ perceptions in controlled conditions, while sentiment analysis provides external validation and confirms the emergent themes and the real-life sentiment orientation of people after the communication.

### Survey data collection and SEM analysis

A self-administered questionnaire is added as a complement to the sentiment data to deliver more insight into the user’s attitudes and experiences. The study subjects were selected from diverse backgrounds, as shown in Table 1. Ethical approval was sought to preserve the integrity of the research process during data collection. The survey period on Questionnaire Star was October 26, 2024, to January 29, 2025, and the survey was conducted using convenience sampling methods, both online and offline. Social media platforms such as Weibo, Reddit, Twitter, and WeChat were used to enhance the recruiting process, and participants were paid upon completing the survey. The study started with 693 responses, of which 62 were ineligible, leaving 632 for final analysis (91.07% response rate). The bilingual questionnaire, translated from English to Chinese, was piloted and split into two parts: demographic information and 31 items for 10 sets of constructs (e.g., AA and PE) from existing scales, using a five-point Likert scale. Data analysis utilized PLS-SEM via SmartPLS 4.1.0.8, evaluating the model’s predictive capabilities to explore AI-powered tool adoption dynamics.

**Table 1:** The profile of the participants.

Variables	Frequency	Percentage (%)
Age		
Under 20	94	14.87
20–29	327	51.74
30–49	175	27.69
Above 50	36	5.70
Gender		
Male	208	32.91
Female	424	67.09
Educational level		
Undergraduate	359	56.80
Master’s	182	28.80
Ph.D./Doctorate	60	9.49
Other	31	4.91
Experience		
Less than 2 years	60	9.49
2–5 years	221	34.97
6–10 years	251	39.72
More than 10 years	100	15.82
Total	632	100

### Sentiment analysis for triangulation and contextualization

**Figure 3:**
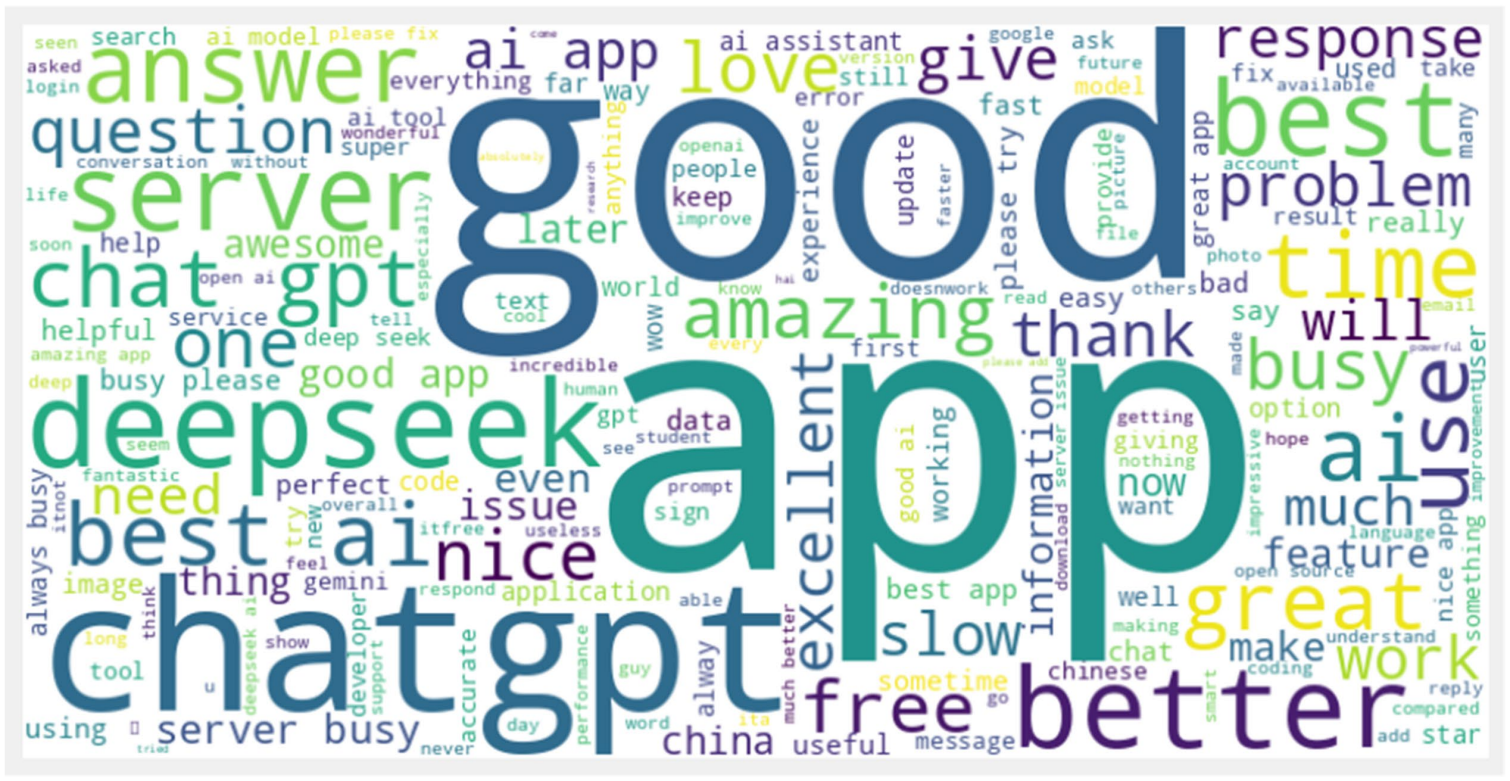
DeepSeek AI cloud.

To contextualize the quantitative relationships identified by the SEM model, a sentiment analysis was performed on 39,310 online reviews and discussions concerning DeepSeek. The main aim of this analysis was to conduct a real-world validity test, using the population’s qualitative sentiment to aid interpretation of the statistical results. In particular, popular keywords and changes in sentiment in unstructured text data provide convincing evidence for the significance or non-significance of the structural paths. Cleaning, tokenization, and sentiment labeling of data were carried out using the NLTK and TextBlob libraries with the help of the data scraped between the date of model launch (January 20, 2025) and October 25, 2025 (see Supplementary material: ‘sentiment analysis’ and ‘web scrape’). This dataset, focused on public discourse around key AI advancements, was filtered using prominent keywords such as “app,” “good,” “ChatGPT,” and “deepseek.” The resulting analysis, shown in Figure 3, reveals the dominance of terms such as “good” and “app” in the online conversation.

## Results

### Questionnaire survey findings

#### The outer measurement model for reliability and validity

The researchers conducted comprehensive experiments with the reflective measurement model, which is used in educational institutions to support AI-based tools. The PLS-SEM algorithm was used to examine indicator loadings in accordance with the guidelines. Sarstedt et al. (2014) further argue that the constructs explained a substantial amount of variance, as the factor loadings exceeded 0.70. The values of AA2 (0.691), EE1 (0.619), and HM3 (0.689) were observed and retained, as the overall reliability and validity indicators attained acceptable levels (Table 2). Each indicator showed high levels of statistical reliability (*p* = 0.05) and above 0.70 (Chin, 1998). Cronbach’s alpha and composite reliability (CR) were used to assess internal consistency. The internal consistency of all constructs was above 0.70, as determined according to Hair et al. (2019). Convergent validity was successfully tested, as the Average Variance Extracted (AVE) values exceeded 0.50, thereby confirming the sufficient consistency of the constructs. The complexity of the analysis approach ensures the validity of the research results, enabling the study of the interaction between human and AI tools in language education.

**Table 2:** Reliability and convergent validity of the measurement model.

Constructs	Item	Factor loading	Cronbach’s alpha	Composite reliability (CR)	Average variance extracted (AVE)
AA	AA1	0.745*	0.785	0.858	0.604
	AA2	0.691*			
	AA3	0.904*			
	AA4	0.752*			
BI	BI1	0.930*	0.918	0.948	0.858
	BI2	0.918*			
	BI3	0.930*			
EE	EE1	0.619*	0.810	0.891	0.738
	EE2	0.962*			
	EE3	0.951*			
FC	FC1	0.914*	0.894	0.934	0.825
	FC2	0.920*			
	FC3	0.890*			
HB	HB1	0.943*	0.923	0.951	0.867
	HB2	0.913*			
	HB3	0.937*			
HM	HM1	0.993*	0.874	0.890	0.733
	HM2	0.860*			
	HM3	0.689*			
PE	PE1	0.728*	0.710	0.839	0.636
	PE2	0.879*			
	PE3	0.778*			
PV	PV1	0.894*	0.900	0.937	0.833
	PV2	0.916*			
	PV3	0.928*			
SI	SI1	0.870*	0.879	0.925	0.805
	SI2	0.917*			
	SI3	0.905*			
UB	UB1	0.902*	0.887	0.929	0.814
	UB2	0.907*			
	UB3	0.897*			

*p < 0.05.

Moreover, the Fornell–Larcker criterion was used to assess discriminant validity in the course of researching AI-based tools for adoption in education. This method of discriminating validity can be validated by comparing the construct AVE square root with construct correlations (Fornell and Larcker, 1981). As shown in Table 3, the bolded diagonal values are the square roots of the AVEs and indicate that they are higher than other construct correlations. BI AVE square root value is 0.926, but at the same time, its correlation with EE is 0.186 and FC is 0.310. The findings reveal that each construct has stronger relations with the measurement variables than the other constructs, thereby confirming discriminant validity (Chin, 1998). Nevertheless, some scholars have argued that the Fornell–Larcker criterion yields higher factor loadings, thereby falsifying the structural relationships in models that employ variance-based SEM techniques (Henseler et al., 2015). The authors do not ignore these concerns; their importance demanded the analysis provided to demonstrate that they were robust enough to justify the study’s findings.

**Table 3:** Fornell–Larcker test of discriminant validity.

Constructs	Correlation of the constructs									
	AA	BI	EE	FC	HB	HM	PE	PV	SI	UB
AA	0.777									
BI	−0.202	0.926								
EE	−0.072	0.186	0.859							
FC	0.013	0.310	−0.056	0.908						
HB	0.260	−0.498	−0.180	−0.222	0.931					
HM	0.045	0.033	−0.094	0.022	0.070	0.856				
PE	−0.013	0.186	−0.066	0.061	−0.045	0.042	0.797			
PV	0.018	0.453	0.095	0.118	−0.332	0.039	0.128	0.913		
SI	−0.129	0.484	0.069	0.090	−0.286	0.049	0.079	0.324	0.897	
UB	−0.148	0.533	0.202	0.447	−0.358	−0.043	−0.044	0.205	0.133	0.902

Heterotrait–Monotrait ratio (HTMT) is used to check for the discriminant validity of the construct. The overall HTMT values are below the cut-off value of 0.85 (Table 4), suggesting differences among the constructs (Henseler et al., 2015). The fact that this model is conformant offers evidence of discriminant validity and indicates that all constructs are based on their respective, unique dimensions, with minimal overlap. The strength of this isolation was critical to models investigating the factors influencing the uptake of GenAI models, such as Deepseek, in foreign language classrooms. The utility of the HTMT analysis is to improve the model’s reliability, ensuring that each construct captures what it is intended to capture and, thus, providing meaningful information about the adoption process.

**Table 4:** Heterotrait–Monotrait Ratio (HTMT) of discriminant validity.

Constructs	AA	BI	EE	FC	HB	HM	PE	PV	SI	UB
AA										
BI	0.222									
EE	0.084	0.215								
FC	0.092	0.336	0.083							
HB	0.318	0.535	0.199	0.245						
HM	0.128	0.024	0.119	0.075	0.118					
PE	0.176	0.230	0.104	0.118	0.064	0.046				
PV	0.082	0.491	0.113	0.129	0.364	0.053	0.157			
SI	0.136	0.536	0.086	0.101	0.316	0.065	0.104	0.364		
UB	0.179	0.579	0.223	0.490	0.388	0.051	0.092	0.222	0.148	

### The (inner) structural model analysis

The research evaluated relationships between variables through structural model analysis following the completion of measurement model reliability and validity assessment. The evaluation process began by analyzing the coefficient of determination (*R*^2^) to assess how well the independent variables explain variation in the dependent variable (Shmueli and Koppius, 2011). The guidelines by Chin (1998) indicate that *R*^2^ values between 0.67 and 0.33 represent substantial explanatory power, while values between 0.33 and 0.19 indicate moderate and weak explanatory power, respectively. The explanatory power of BI reached 0.454 in this research, and UB achieved 0.379, indicating substantial strength. Using AI-powered language-learning tools, the model successfully predicts 45.4% of users’ BI and 37.9% of their UB behavior. The model fit indices demonstrate a good fit according to Henseler et al. (2016a,b), as the standardized root mean square residual (SRMR) value (0.055) is below 0.08. The findings from PLS-SEM demonstrate that this model successfully predicts user actions regarding AI-powered educational technology and their behavioral intentions towards it.

**Figure 4:**
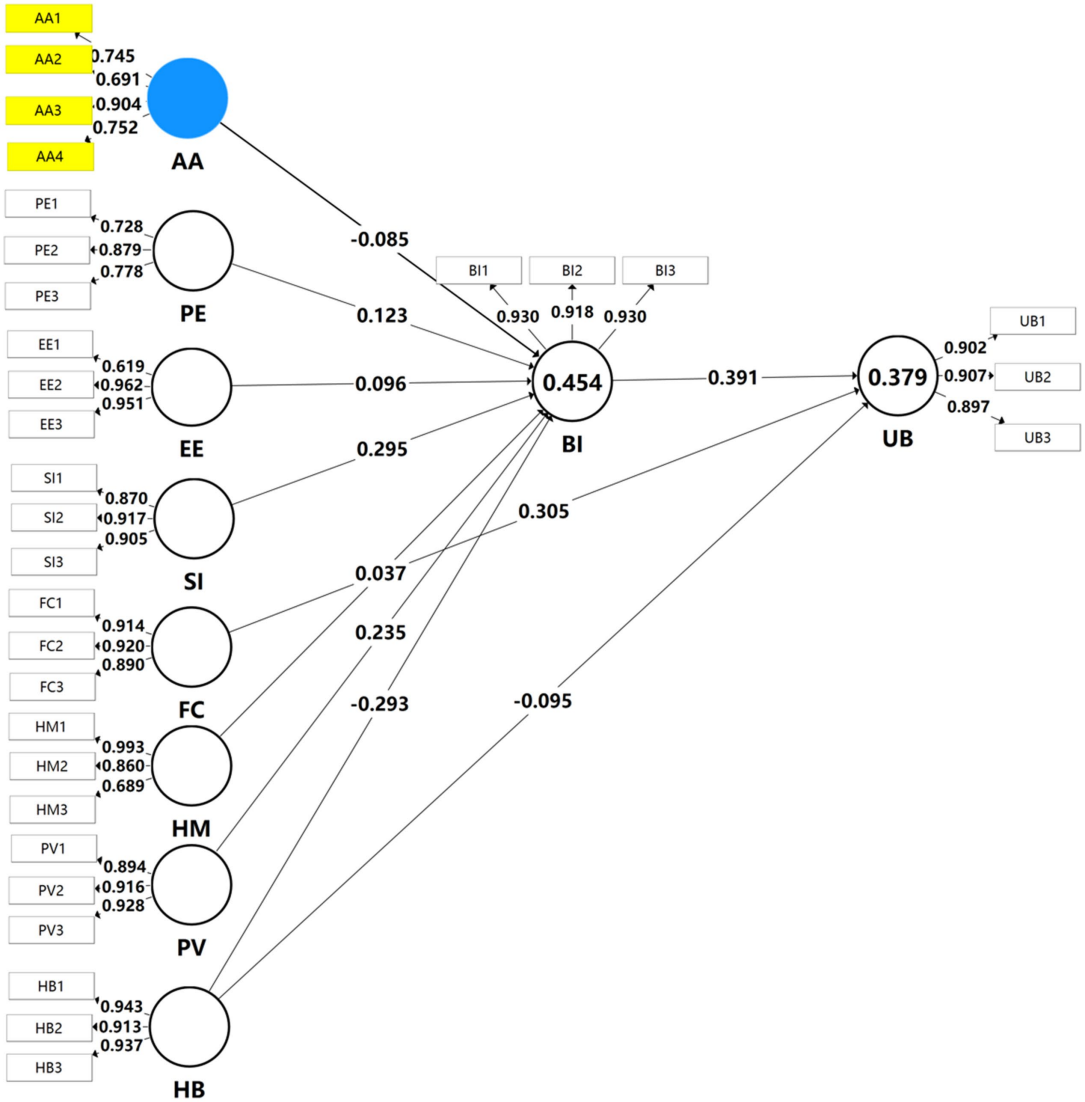
The established PLS-SEM.

The statistical tests confirmed the significance of 9 of the 10 proposed paths in the study. The analysis demonstrates that AA negatively predicts BI (*β* = −0.085, *p* = 0.004), thus validating H1. Similarly, PE significantly impacts BI (*β* = 0.123, *p* < 0.001), supporting H2. EE also positively affects BI (*β* = 0.096, *p* = 0.002), confirming H3. SI was found to be a strong positive predictor of BI (*β* = 0.295, *p* < 0.001), confirming H4. The research confirmed H5 through the positive relationship between FC and UB (*β* = 0.305, *p* < 0.001). However, HM did not substantially impact BI (*β* = 0.037, *p* = 0.240), leading to the rejection of H6. The study demonstrates that PV positively affects BI (*β* = 0.235, *p* < 0.001), validating H7. The results show that HB negatively influences both BI (*β* = −0.293, *p* < 0.001) and UB (*β* = −0.095, *p* = 0.006), confirming both H8 and H9. Finally, BI was found to directly impact UB with high statistical significance (*β* = 0.391, *p* < 0.001), supporting H10. Table 5 and Figure 4 presents the final established model.

**Table 5:** The results of hypothesis testing.

No.	Path	Path coefficient	Confidence interval		T statistics	*p-*values	Results
			2.5%	97.5%			
H1	AA → BI	−0.085	−0.140	−0.023	2.866	0.004	Accepted
H2	PE → BI	0.123	0.064	0.170	4.516	0.000	Accepted
H3	EE → BI	0.096	0.042	0.166	3.084	0.002	Accepted
H4	SI → BI	0.295	0.214	0.363	8.158	0.000	Accepted
H5	FC → UB	0.305	0.236	0.360	9.494	0.000	Accepted
H6	HM → BI	0.037	−0.041	0.089	1.176	0.240	Rejected
H7	PV → BI	0.235	0.174	0.297	4.516	0.000	Accepted
H8	HB → BI	−0.293	−0.354	−0.228	9.087	0.000	Accepted
H9	HB → UB	−0.095	−0.158	−0.025	2.775	0.006	Accepted
H10	BI → UB	0.391	0.329	0.454	12.229	0.000	Accepted

### Mediating analysis

The mediation analysis results in Table 6 reveal significant indirect effects on UB through BI, with EE (*β* = 0.038, 95% CI [0.016, 0.065]), HB (*β* = −0.115, 95% CI [−0.144, −0.088]), PE (*β* = 0.048, 95% CI [0.027, 0.068]), PV (*β* = 0.092, 95% CI [0.061, 0.130]), and SI (*β* = 0.115, 95% CI [0.087, 0.151]) all exhibiting statistically significant mediation paths (*p* < 0.01). Furthermore, a significant adverse indirect effect was found for AA (*β* = −0.033, 95% CI [−0.057, −0.012], *p* < 0.01). Results support the view that the influence of BI on UB is a complex phenomenon: While EE, PE, PV, and SI have a positive effect on UB, BI also negatively affects UB by incorporating the effects of HB and AA. The findings highlight BI as an important mediator of UB, and SI demonstrated the most substantial positive indirect effect.

**Table 6:** Significant mediating effects of the research model.

Path	Indirect effect	Standard deviation (STDEV)	Confidence interval		T statistics	*p-*values
			2.5%	97.5%		
AA → BI → UB	−0.033	0.012	−0.057	−0.012	2.717	0.007
EE → BI → UB	0.038	0.013	0.016	0.065	2.973	0.003
HB → BI → UB	−0.115	0.014	−0.144	−0.088	8.246	0.000
PE → BI → UB	0.048	0.011	0.027	0.068	4.523	0.000
PV → BI → UB	0.092	0.017	0.061	0.130	5.336	0.000
SI → BI → UB	0.115	0.016	0.087	0.151	7.073	0.000

### Sentiment analysis findings

**Figure 5:**
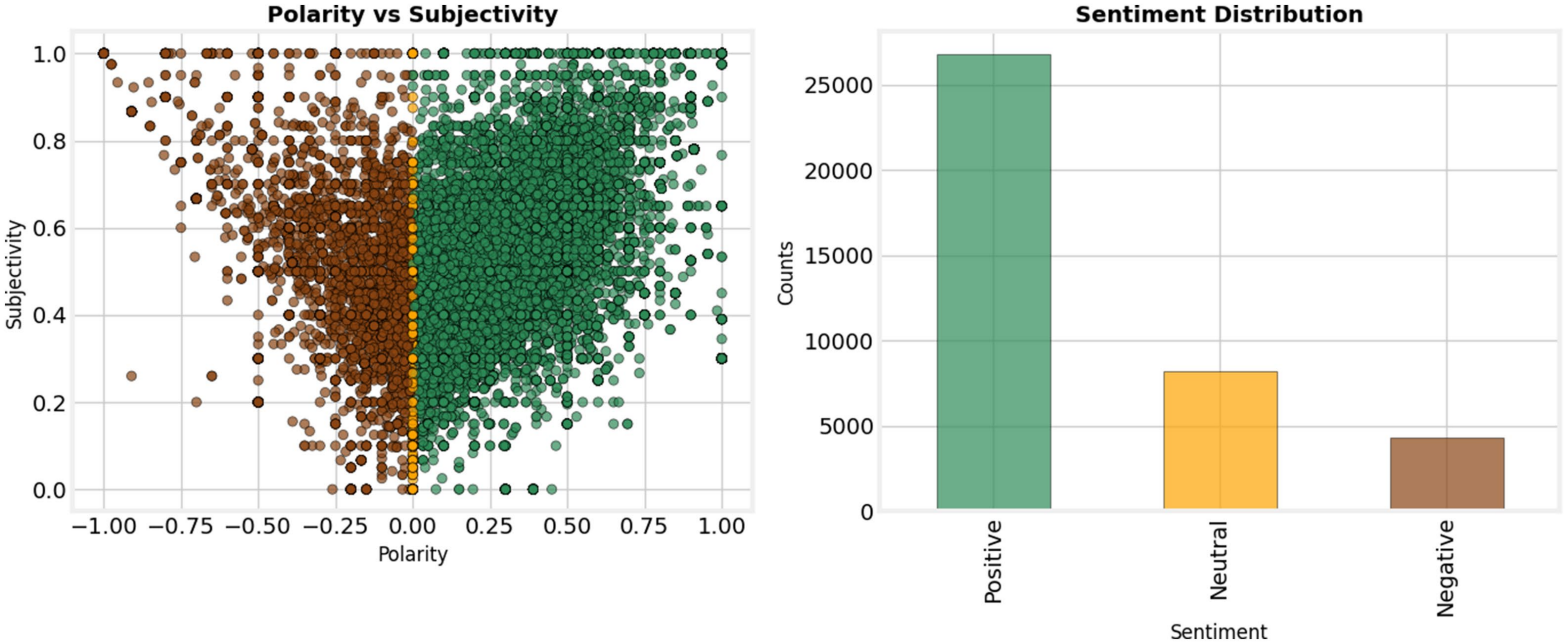
Sentiment distribution and polarity-subjectivity relationship in text data.

Though SEM analysis can quantitatively establish the structural relationships between constructs, sentiment analysis provides a critical qualitative basis for understanding them. The very high rating (68.1%) for AI tools such as DeepSeek could have contributed to the strong positive effect of PE, SI, and in the SEM model. On the other hand, negative sentiment (11.0%) and neutral attitude (20.9%) are triangulated with negative correlations with anxiety and habit, as shown in Figure 5. These results imply that users’ fears and pre-conceived patterns of behavior are barriers to take-up that must be considered. More importantly, the sentiment analysis goes beyond the binary positive–negative framing of the choices to show the complex subjective space of adoption choices, where even positive users are ambivalent. This combination of subjective user attitudes and the results of the structural models gives the impression of how subjective user attitudes complement the latter, which would not provide such a detailed account of the dynamics of adoption.

## Discussion

The results of the extended UTAUT2 model were validated empirically and provide granular and more data-driven psychological insights regarding GenAI adoption in foreign language education. The strong measurement and structural models confirmed that important constructs especially SI and PE are good positive predictors of BI and BI has a good predictive relationship with usage behavior. However, the negative trajectories that emerged (habit and anxiety) and the non-significant HM effect suggest a complex adoption profile that is beyond technological convenience. These findings pave the way for a more general discourse on the entangled flock of the user’s psychology, teaching practice and the accelerated adoption of intelligent technologies in the contemporary teaching setting, in the context of complementary sentiment analysis, which points to a generally positive attitude towards the user, but with nuances.

### Key psychological drivers of GenAI adoption

AI Anxiety was found to be a strong negative antecedent of BI. While recent discussions in the literature on AI anxiety have conceptualised anxiety in terms of technophobia, this finding has important implications. It is consistent with literature suggesting that AI anxiety is a multidimensional concept rooted in perceived job loss, algorithmic black boxes, and the loss of human agency (Kim J. J. H. et al., 2025; Li and Huang, 2020; Zhan et al., 2024). The predominant negative channel shows that, for education experts, perceived risks of GenAI use (undervaluing pedagogical skills and leading to an overreliance on untrustworthy systems) outweigh its usefulness, hindering adoption. This conclusion abandons optimistic, technological-deterministic narratives in the literature on the adoption of educational technology, rooted in PE, and rejects the overwhelming role of radical psychological discomfort. This is particularly so for the discipline of foreign languages teaching, whose close relationship to human communication and cultural subtlety is threatened by the loss of some pedagogical or formative functions to a ‘black box’ algorithm.

Although social Influence has been validated as a powerful theoretical construct, it needs to be critically examined in relation to its operationalisation and its underlying theory. The results supported SI as the behavioral variable affecting adopter behavior, similar to research on the AI tutoring system and the Massive Open Online Course (MOOC) (Al-Abdullatif et al., 2023). While the result is consistent with the original UTAUT proposition, the opinions of significant others do affect people; the complexity of Influence may be blurred in technologically novel areas of education, such as GenAI. Even if the coefficient of path is significant, it does not necessarily mean the individual is normatively compliant. Still, one may be picking up something more subtle: “informational social influence,” in which, in the face of substantial certainty about the pedagogical quality and ethical implications of AI, educators look to the advice of their peers and institutional authorities as heuristics for quality and legitimacy. The result is counterintuitive on a prima facie basis, in that the power of SI is not related to passive conformity, but is the product of an active, cognitive process of eliminating uncertainty in an emergent domain. Moreover, the finding constitutes an important counterargument to a growing literature finding that SI is a weaker predictor of individual, voluntary technologies, and implies a more substantial effect due to the high-stakes, collaborative nature of educational settings.

Habit exerts a paradoxical, counterintuitive influence within the extended UTAUT2 model, as these findings reveal a significant negative impact on both behavioral intention and use behavior. The rationale for this discrepancy may be in the novel and cognitively demanding nature of GenAI tools in an educational context, rather than inducing automaticity, the initial creation of a “habit” may be a conscious, effortful coping strategy that users consciously associate with the cognitive load and potential anxiety associated with mastering a cognitively complex new technology. As a result, this measured “habit” could be a proxy for the disruption to established learning routines and, thus, psychological reactance to complete adoption. This appears to imply that, at least with transformative technologies such as GenAI, the way habit is facilitative may be reversed in the early phases of adoption, in which its presence is indicative not of ease of use but of a conscious effort to incorporate the technology into one’s pedagogical or learning workflow.

Performance Expectancy was identified as a significant but relatively weak predictor of BI in this research, raising questions about the critical engagement with the literature. While the outcome supports the fundamental UTAUT2 result that P.U. is a driver of technology adoption (Chun and Yunus, 2023; Kim S. et al., 2025; Malarvizhi et al., 2022), the relatively low beta coefficient indicates a more complex picture for GenAI in L.L. This reduced impact may call into question the universal dominance of PE, suggesting that in the case of advanced, generative technologies its impact may be influenced or even replaced by other psychological factors, such as a “fear of deskilling” human language or doubts in the reliability of the given technology in terms of culturally and grammatically correct answers. This mediated effect could relate to the task itself; while learners will be able to see the potential of GenAI to be efficient (instant translation, grammar correction), they might also be sceptical of GAn’s pedagogical aims in terms of building up deep and nuanced communicative competence (much less than more deterministic tools such as grammar checkers, or digital flashcards). Thus, our findings is a critical addition to the literature in that it argues that the performance benefits precipitated through GenAI are offset by a more nuanced set of evaluative criteria by adopters of GenAI in educational contexts.

#### Non-significant factors

Hedonic Motivation addresses a complex adoption that is beyond the technology’s usefulness; its irrelevance to BI, as applied to GenAI in learning a foreign language, warrants critical attention and should be compared with the current literature. This is possibly because the task and user perception are as they are; compared to casual technologies, GenAI adoption for structured learning is likely framed by learners as a “serious” instrumental aid for proficiency, and hedonic benefits are dominated by Utilitarian constructs such as PE and PV. Moreover, as GenAI is still in its early integration stage, initial user interactions may not be about cognitive load and novelty exploration but about fun, and hedonic factors may be inseparable from good, personalized learning outcomes, which can lead to the loss of statistical significance of HM when you control for other powerful predictors in the model.

#### Mediating factors

Mediating analysis shows BI to be a key, but incomplete, explanatory mechanism in the adoption of GenAI for language learning, which forces a critical re-examination of the sufficiency of the UTAUT2 framework. While the significant positive indirect effects of PE, EE, and SI via BI are consistent with the canonical postulations of UTAUT2 (Liu et al., 2025; Pilotti et al., 2025), the intense negative mediation by habit and anxiety reveals a more complex psychological picture. The negative strong path for HB paradoxically implies that established learning routines may actively prevent the formation of intentions to adopt innovative GenAI tools. This finding challenges the often linear, optimistic assumptions of models of technology adoption and resonates with the literature on cognitive inertia in educational contexts (Sackstein et al., 2023). Similarly, the negative mediation of AA highlights the fact that the adverse effect of anxiety acts to a large degree by inhibiting the very intention to engage, which is often ignored in broader correlational research. However, the fact that these mediators, although important, are not responsible for the full extent of the relations (as direct effects likely remain) suggests that BI is a partial mediator and that other factors (not measured in the research, e.g., prior experience, specific pedagogical design) may exert direct influences on usage behavior.

### Sentiment analysis: public perception of GenAI

Results from public sentiment analysis show widespread positive attitudes toward DeepSeek GenAI tools, as societal expressions indicate expectations that these tools will provide educational value consistent with survey findings on usefulness. A significant number of neutral and negative comments about the tool point to ongoing issues in the ethics of algorithmic bias and overutilization, as broader critiques found in Calvo et al. (2020). Two-sided public reception of AI reflects the necessity of responsible implementation of next-generation AI development and overcoming existing skepticism. In the long run, language education will adopt GenAI as developers devise ways to ensure continuous innovation and establish ethical standards that build trust between learners and educators.

### Theoretical contributions

This article makes an important theoretical contribution by empirically demonstrating that AI Anxiety is a critical, direct negative predictor of BI in the adoption of GenAI in foreign language education, thereby extending the scope of UTAUT2. Whereas current models of technology acceptance center on enabling or facilitating constructs, this research is theoretically important because it shows that an AI-specific emotional barrier, AI-anxiety, is a strong and unique barrier to adoption, distinct from other constructs such as habit, performance expectancy, etc. It confirms a significant negative path from AA to BI and a significant adverse indirect effect from AA to BI on use behavior, thus providing strong empirical support for treating emotional apprehensions as a key determinant, alongside cognitive and social influences, in the adoption model of advanced AI technologies. This makes AI anxiety not just a peripheral concern but a central theoretical construct that must be considered to fully understand and anticipate users’ acceptance of AI in educational settings where complex AI tools are used.

### Pedagogical implications

Based on the solid results of this extended UTAUT2 model analysis, we can draw several important pedagogical implications for incorporating GenAI into the teaching of foreign languages. The substantial positive impact of PE, EE, and, in particular, SI on BI also underscores the importance of instructors not only showing how GenAI tools effectively improve learning outcomes and are easy to use, but also consciously creating a positive normative environment for adoption. This can be done by securing faculty-wide buy-in, running successful peer implementations through workshops, and forming communities of practice to build momentum. On the other hand, the strong adverse effect of Anxiety and Habit suggests that pedagogical interventions need to be proactive to counter resistance, by planning low-stakes scaffolded activities to build familiarity and demystify the technology to lessen anxiety, and to show them how to incorporate these new tools into, or replace, their old study practices. Since FC was one of the direct promoters of UB, institutions must build robust technological infrastructure and provide readily available support. In conclusion, these findings promote a holistic approach to tool adoption that pairs messengers of persuasive communication about the tool’s value with hands-on training and robust institutional support to ease psychological barriers and align with institutional culture for technology innovation in the language classroom.

Practically, the results will illustrate global knowledge of artificial intelligence technology adoption behavior that transcends regional boundaries in foreign language teaching, which will undergo a fundamental change once Artificial General Intelligence is realized, enabling the generation of personalized, flexible content. The deployment of DeepSeek requires integrating DeepSeek into the system and understanding user and public acceptance, as well as the influence of the adoption process. The research reveals that institutions and policy-makers need to adopt strategies to boost user trust in the system by introducing peer-based recommendation policies and low-cost implementation programs. AI systems require long-term success predicated on three key factors: open functions, improved data security, and the absolute trustworthiness of each sequence of processes. The achievement of a possible AGI depends on society overcoming existing ethical challenges and institutional barriers; otherwise, it will exacerbate inequality in its education system.

### Ethical implications

The ethical implications of this study on the psychological determinants of GenAI adoption in foreign language education include responsible use, data privacy, and potential impact on human roles of learning and teaching. While the extended UTAUT2 model is an effective predictor of user behavior towards artificial intelligence-powered educational tools, there are ethical concerns associated with data collection and analysis involving participants using survey instruments, such as the importance of informed consent and the protection of individual information. Furthermore, the use of GenAI in language learning also raises questions about academic integrity, overreliance on automated systems, and the role of human educators. As the implementation of biased algorithms or unequal access to technology could worsen existing educational inequalities, it is important to ensure fairness, transparency, and accountability in the use of AI in education. Therefore, although the research will add to the knowledge base on the adoption of GenAI, the study highlights the ethical need to balance technological innovation and human agency, privacy, and equitable access in education.

## Conclusion

### Major findings

According to this extended UTAUT2 analysis, the behavioral intention to adopt GenAI to learn foreign languages is significantly and positively influenced by SI, PE, and EE. In contrast, anxiety, as well as existing learning habits, end up having significant negative influences. The model shows a significant predictive power. 45.4% of the variance in BI and 37.9% of the variance in UB are explained. 45.4% of the variance in behavioral intention and 37.9% of the variance in use behavior were explained, with behavioral intention as an important mediator between the psychological determinants and actual use. The rejection of the hedonic motivation hypothesis implies that the major driver of adoption is utilitarian rather than hedonic. Importantly, sentiment analysis is used to triangulate these quantitative findings, indicating that despite a generally positive attitude towards AI tools, underlying apprehensions and well-established behavioral patterns, complex dynamics of adoption exist that go beyond acceptance-rejection binaries.

### Limitations

The important insights into the predictors of GenAI acceptance in language learning from a psychological perspective can prompt us to reflect on several research limitations. First, online recruitment has used Weibo in addition to Reddit, Twitter, and WeChat to recruit participants, which can introduce self-selection bias, as the results of assessments are skewed toward the technology-savvy who adopt AI recruitment. Second, including participants from diverse backgrounds and underrepresented groups would also improve the generalizability of research findings. Third, the horizontal research methodology has its shortcomings when it comes to determining cause and effect, as the behavior change can only be measured by following groups over a long period of time. Also, the sentiment analysis approach was based on Twitter content and did not directly include contributions from educators and learners; qualitative interview data were required for a more granular analysis.

### Future research directions

Future studies require improving the current research through three means: observing changes in user behavior and acceptance trends over time, increasing their geographical reach to include diverse populations and demographic areas, and implementing qualitative research tools to deepen understanding of user interactions. Future AI research needs to analyze morality in data protection alongside algorithmic discrimination and the equality of educational resources to help establish moral AI regulations. Understanding teacher training needs, institutional support, and curriculum models for AI adoption will help to offer practical guidance to authorities and teaching personnel. Researching the operation of different AI models across various educational settings would yield practical methods for improving AI integration across multiple learning settings. As GenAI continues to evolve, interdisciplinary collaboration will be key to realizing its potential in classrooms around the world equitably and effectively.

## Data Availability

The original contributions presented in the study are included in the article/Supplementary material, further inquiries can be directed to the corresponding author. Materials related to the article can be graciously accessed through the following link: https://osf.io/tascq/overview?view_only=0df888ec9dbc4be8b18a0db831719c69.
