# Supplementary material for: Psychological determinants of GenAI adoption for foreign language education: an extended UTAUT2 model and sentiment analysis approach
Source: Front Psychol. 2026 Jan 9;16:1622926. doi: 10.3389/fpsyg.2025.1622926 (PMC12827767; doi:10.3389/fpsyg.2025.1622926)

# Import the libraries

# Intalling NLP Libraries
!pip install tweepy
!pip install pyspellchecker
!pip install scattertext
!pip install nltk
!pip install kagglehub

Requirement already satisfied: tweepy in /usr/local/lib/python3.11/dist-packages (4.16.0)
Requirement already satisfied: oauthlib<4,>=3.2.0 in /usr/local/lib/python3.11/dist-packages (from tweepy) (3.3.1)
Requirement already satisfied: requests<3,>=2.27.0 in /usr/local/lib/python3.11/dist-packages (from tweepy) (2.32.5)
Requirement already satisfied: requests-oauthlib<3,>=1.2.0 in /usr/local/lib/python3.11/dist-packages (from tweepy) (2.0.0)
Requirement already satisfied: charset_normalizer<4,>=2 in /usr/local/lib/python3.11/dist-packages (from requests<3,>=2.27.0->tweepy) (3.4.3)
Requirement already satisfied: idna<4,>=2.5 in /usr/local/lib/python3.11/dist-packages (from requests<3,>=2.27.0->tweepy) (3.10)
Requirement already satisfied: urllib3<3,>=1.21.1 in /usr/local/lib/python3.11/dist-packages (from requests<3,>=2.27.0->tweepy) (2.5.0)
Requirement already satisfied: certifi>=2017.4.17 in /usr/local/lib/python3.11/dist-packages (from requests<3,>=2.27.0->tweepy) (2025.8.3)
Collecting pyspellchecker
 Downloading pyspellchecker-0.8.3-py3-none-any.whl.metadata (9.5 kB)
Downloading pyspellchecker-0.8.3-py3-none-any.whl (7.2 MB)
━━━━━━━━━━━━━━━━━━━━━━━━━━━━━━━━━━━━━━━━ 7.2/7.2 MB 69.4 MB/s eta 0:00:00:00:010:01
etadata (581 bytes)
Requirement already satisfied: numpy>=1.2.6 in /usr/local/lib/python3.11/dist-packages (from scattertext) (1.26.4)
Collecting scipy<1.14.0,>=1.7.0 (from scattertext)
 Downloading scipy-1.13.1-cp311-cp311-manylinux_2_17_x86_64.manylinux2014_x86_64.whl.metadata (60 kB)
━━━━━━━━━━━━━━━━━━━━━━━━━━━━━━━━━━━━━━━━ 60.6/60.6 kB 2.1 MB/s eta 0:00:00
 scattertext)
 Downloading scikit_learn-1.7.2-cp311-cp311-manylinux2014_x86_64.manylinux_2_17_x86_64.whl.metadata (11 kB)
Requirement already satisfied: pandas>=2.0.0 in /usr/local/lib/python3.11/dist-packages (from scattertext) (2.2.3)
Requirement already satisfied: statsmodels>=0.14.1 in /usr/local/lib/python3.11/dist-packages (from scattertext) (0.14.5)
Collecting flashtext>=2.7 (from scattertext)
 Downloading flashtext-2.7.tar.gz (14 kB)
 Preparing metadata (setup.py) ... ent already satisfied: gensim>=4.0.0 in /usr/local/lib/python3.11/dist-packages (from scattertext) (4.3.3)
Requirement already satisfied: spacy>=3.2 in /usr/local/lib/python3.11/dist-packages (from scattertext) (3.8.7)
Requirement already satisfied: tqdm>=4.0 in /usr/local/lib/python3.11/dist-packages (from scattertext) (4.67.1)
Requirement already satisfied: smart-open>=1.8.1 in /usr/local/lib/python3.11/dist-packages (from gensim>=4.0.0->scattertext) (7.3.0.post1)
Requirement already satisfied: mkl_fft in /usr/local/lib/python3.11/dist-packages (from numpy>=1.2.6->scattertext) (1.3.8)
Requirement already satisfied: mkl_random in /usr/local/lib/python3.11/dist-packages (from numpy>=1.2.6->scattertext) (1.2.4)
Requirement already satisfied: mkl_umath in /usr/local/lib/python3.11/dist-packages (from numpy>=1.2.6->scattertext) (0.1.1)
Requirement already satisfied: mkl in /usr/local/lib/python3.11/dist-packages (from numpy>=1.2.6->scattertext) (2025.2.0)
Requirement already satisfied: tbb4py in /usr/local/lib/python3.11/dist-packages (from numpy>=1.2.6->scattertext) (2022.2.0)
Requirement already satisfied: mkl-service in /usr/local/lib/python3.11/dist-packages (from numpy>=1.2.6->scattertext) (2.4.1)
Requirement already satisfied: python-dateutil>=2.8.2 in /usr/local/lib/python3.11/dist-packages (from pandas>=2.0.0->scattertext) (2.9.0.post0)
Requirement already satisfied: pytz>=2020.1 in /usr/local/lib/python3.11/dist-packages (from pandas>=2.0.0->scattertext) (2025.2)
Requirement already satisfied: tzdata>=2022.7 in /usr/local/lib/python3.11/dist-packages (from pandas>=2.0.0->scattertext) (2025.2)
Requirement already satisfied: joblib>=1.2.0 in /usr/local/lib/python3.11/dist-packages (from scikit-learn>=1.4->scattertext) (1.5.2)
Requirement already satisfied: threadpoolctl>=3.1.0 in /usr/local/lib/python3.11/dist-packages (from scikit-learn>=1.4->scattertext) (3.6.0)
Requirement already satisfied: spacy-legacy<3.1.0,>=3.0.11 in /usr/local/lib/python3.11/dist-packages (from spacy>=3.2->scattertext) (3.0.12)
Requirement already satisfied: spacy-loggers<2.0.0,>=1.0.0 in /usr/local/lib/python3.11/dist-packages (from spacy>=3.2->scattertext) (1.0.5)
Requirement already satisfied: murmurhash<1.1.0,>=0.28.0 in /usr/local/lib/python3.11/dist-packages (from spacy>=3.2->scattertext) (1.0.13)
Requirement already satisfied: cymem<2.1.0,>=2.0.2 in /usr/local/lib/python3.11/dist-packages (from spacy>=3.2->scattertext) (2.0.11)
Requirement already satisfied: preshed<3.1.0,>=3.0.2 in /usr/local/lib/python3.11/dist-packages (from spacy>=3.2->scattertext) (3.0.10)
Requirement already satisfied: thinc<8.4.0,>=8.3.4 in /usr/local/lib/python3.11/dist-packages (from spacy>=3.2->scattertext) (8.3.6)
Requirement already satisfied: wasabi<1.2.0,>=0.9.1 in /usr/local/lib/python3.11/dist-packages (from spacy>=3.2->scattertext) (1.1.3)
Requirement already satisfied: srsly<3.0.0,>=2.4.3 in /usr/local/lib/python3.11/dist-packages (from spacy>=3.2->scattertext) (2.5.1)
Requirement already satisfied: catalogue<2.1.0,>=2.0.6 in /usr/local/lib/python3.11/dist-packages (from spacy>=3.2->scattertext) (2.0.10)
Requirement already satisfied: weasel<0.5.0,>=0.1.0 in /usr/local/lib/python3.11/dist-packages (from spacy>=3.2->scattertext) (0.4.1)
Requirement already satisfied: typer<1.0.0,>=0.3.0 in /usr/local/lib/python3.11/dist-packages (from spacy>=3.2->scattertext) (0.16.0)
Requirement already satisfied: requests<3.0.0,>=2.13.0 in /usr/local/lib/python3.11/dist-packages (from spacy>=3.2->scattertext) (2.32.5)
Requirement already satisfied: pydantic!=1.8,!=1.8.1,<3.0.0,>=1.7.4 in /usr/local/lib/python3.11/dist-packages (from spacy>=3.2->scattertext) (2.12.0a1)
Requirement already satisfied: jinja2 in /usr/local/lib/python3.11/dist-packages (from spacy>=3.2->scattertext) (3.1.6)
Requirement already satisfied: setuptools in /usr/local/lib/python3.11/dist-packages (from spacy>=3.2->scattertext) (75.2.0)
Requirement already satisfied: packaging>=20.0 in /usr/local/lib/python3.11/dist-packages (from spacy>=3.2->scattertext) (25.0)
Requirement already satisfied: langcodes<4.0.0,>=3.2.0 in /usr/local/lib/python3.11/dist-packages (from spacy>=3.2->scattertext) (3.5.0)
Requirement already satisfied: patsy>=0.5.6 in /usr/local/lib/python3.11/dist-packages (from statsmodels>=0.14.1->scattertext) (1.0.1)
Requirement already satisfied: language-data>=1.2 in /usr/local/lib/python3.11/dist-packages (from langcodes<4.0.0,>=3.2.0->spacy>=3.2->scattertext) (1.3.0)
Requirement already satisfied: annotated-types>=0.6.0 in /usr/local/lib/python3.11/dist-packages (from pydantic!=1.8,!=1.8.1,<3.0.0,>=1.7.4->spacy>=3.2->scattertext) (0.7.0)
Requirement already satisfied: pydantic-core==2.37.2 in /usr/local/lib/python3.11/dist-packages (from pydantic!=1.8,!=1.8.1,<3.0.0,>=1.7.4->spacy>=3.2->scattertext) (2.37.2)
Requirement already satisfied: typing-extensions>=4.14.1 in /usr/local/lib/python3.11/dist-packages (from pydantic!=1.8,!=1.8.1,<3.0.0,>=1.7.4->spacy>=3.2->scattertext) (4.15.0)
Requirement already satisfied: typing-inspection>=0.4.0 in /usr/local/lib/python3.11/dist-packages (from pydantic!=1.8,!=1.8.1,<3.0.0,>=1.7.4->spacy>=3.2->scattertext) (0.4.1)
Requirement already satisfied: six>=1.5 in /usr/local/lib/python3.11/dist-packages (from python-dateutil>=2.8.2->pandas>=2.0.0->scattertext) (1.17.0)
Requirement already satisfied: charset_normalizer<4,>=2 in /usr/local/lib/python3.11/dist-packages (from requests<3.0.0,>=2.13.0->spacy>=3.2->scattertext) (3.4.3)
Requirement already satisfied: idna<4,>=2.5 in /usr/local/lib/python3.11/dist-packages (from requests<3.0.0,>=2.13.0->spacy>=3.2->scattertext) (3.10)
Requirement already satisfied: urllib3<3,>=1.21.1 in /usr/local/lib/python3.11/dist-packages (from requests<3.0.0,>=2.13.0->spacy>=3.2->scattertext) (2.5.0)
Requirement already satisfied: certifi>=2017.4.17 in /usr/local/lib/python3.11/dist-packages (from requests<3.0.0,>=2.13.0->spacy>=3.2->scattertext) (2025.8.3)
Requirement already satisfied: wrapt in /usr/local/lib/python3.11/dist-packages (from smart-open>=1.8.1->gensim>=4.0.0->scattertext) (1.17.2)
Requirement already satisfied: blis<1.4.0,>=1.3.0 in /usr/local/lib/python3.11/dist-packages (from thinc<8.4.0,>=8.3.4->spacy>=3.2->scattertext) (1.3.0)
Requirement already satisfied: confection<1.0.0,>=0.0.1 in /usr/local/lib/python3.11/dist-packages (from thinc<8.4.0,>=8.3.4->spacy>=3.2->scattertext) (0.1.5)
INFO: pip is looking at multiple versions of thinc to determine which version is compatible with other requirements. This could take a while.
Collecting thinc<8.4.0,>=8.3.4 (from spacy>=3.2->scattertext)
 Downloading thinc-8.3.4-cp311-cp311-manylinux_2_17_x86_64.manylinux2014_x86_64.whl.metadata (15 kB)
Collecting blis<1.3.0,>=1.2.0 (from thinc<8.4.0,>=8.3.4->spacy>=3.2->scattertext)
 Downloading blis-1.2.1-cp311-cp311-manylinux_2_17_x86_64.manylinux2014_x86_64.whl.metadata (7.4 kB)
Requirement already satisfied: click>=8.0.0 in /usr/local/lib/python3.11/dist-packages (from typer<1.0.0,>=0.3.0->spacy>=3.2->scattertext) (8.3.0)
Requirement already satisfied: shellingham>=1.3.0 in /usr/local/lib/python3.11/dist-packages (from typer<1.0.0,>=0.3.0->spacy>=3.2->scattertext) (1.5.4)
Requirement already satisfied: rich>=10.11.0 in /usr/local/lib/python3.11/dist-packages (from typer<1.0.0,>=0.3.0->spacy>=3.2->scattertext) (14.1.0)
Requirement already satisfied: cloudpathlib<1.0.0,>=0.7.0 in /usr/local/lib/python3.11/dist-packages (from weasel<0.5.0,>=0.1.0->spacy>=3.2->scattertext) (0.21.1)
Requirement already satisfied: MarkupSafe>=2.0 in /usr/local/lib/python3.11/dist-packages (from jinja2->spacy>=3.2->scattertext) (3.0.2)
Requirement already satisfied: intel-openmp<2026,>=2024 in /usr/local/lib/python3.11/dist-packages (from mkl->numpy>=1.2.6->scattertext) (2024.2.0)
Requirement already satisfied: tbb==2022.* in /usr/local/lib/python3.11/dist-packages (from mkl->numpy>=1.2.6->scattertext) (2022.2.0)
Requirement already satisfied: tcmlib==1.* in /usr/local/lib/python3.11/dist-packages (from tbb==2022.*->mkl->numpy>=1.2.6->scattertext) (1.4.0)
Requirement already satisfied: intel-cmplr-lib-rt in /usr/local/lib/python3.11/dist-packages (from mkl_umath->numpy>=1.2.6->scattertext) (2024.2.0)
Requirement already satisfied: intel-cmplr-lib-ur==2024.2.0 in /usr/local/lib/python3.11/dist-packages (from intel-openmp<2026,>=2024->mkl->numpy>=1.2.6->scattertext) (2024.2.0)
Requirement already satisfied: marisa-trie>=1.1.0 in /usr/local/lib/python3.11/dist-packages (from language-data>=1.2->langcodes<4.0.0,>=3.2.0->spacy>=3.2->scattertext) (1.2.1)
Requirement already satisfied: markdown-it-py>=2.2.0 in /usr/local/lib/python3.11/dist-packages (from rich>=10.11.0->typer<1.0.0,>=0.3.0->spacy>=3.2->scattertext) (4.0.0)
Requirement already satisfied: pygments<3.0.0,>=2.13.0 in /usr/local/lib/python3.11/dist-packages (from rich>=10.11.0->typer<1.0.0,>=0.3.0->spacy>=3.2->scattertext) (2.19.2)
Requirement already satisfied: mdurl~=0.1 in /usr/local/lib/python3.11/dist-packages (from markdown-it-py>=2.2.0->rich>=10.11.0->typer<1.0.0,>=0.3.0->spacy>=3.2->scattertext) (0.1.2)
Downloading scattertext-0.2.2-py3-none-any.whl (9.4 MB)
━━━━━━━━━━━━━━━━━━━━━━━━━━━━━━━━━━━━━━━━ 9.4/9.4 MB 16.8 MB/s eta 0:00:0000:0100:01
anylinux2014_x86_64.manylinux_2_17_x86_64.whl (9.7 MB)
━━━━━━━━━━━━━━━━━━━━━━━━━━━━━━━━━━━━━━━━ 9.7/9.7 MB 107.2 MB/s eta 0:00:0000:0100:01
anylinux_2_17_x86_64.manylinux2014_x86_64.whl (38.6 MB)
━━━━━━━━━━━━━━━━━━━━━━━━━━━━━━━━━━━━━━━━ 38.6/38.6 MB 51.9 MB/s eta 0:00:00:00:0100:01
anylinux_2_17_x86_64.manylinux2014_x86_64.whl (3.9 MB)
━━━━━━━━━━━━━━━━━━━━━━━━━━━━━━━━━━━━━━━━ 3.9/3.9 MB 85.0 MB/s eta 0:00:00:00:01
anylinux_2_17_x86_64.manylinux2014_x86_64.whl (11.7 MB)
━━━━━━━━━━━━━━━━━━━━━━━━━━━━━━━━━━━━━━━━ 11.7/11.7 MB 115.2 MB/s eta 0:00:0000:010:01
e=flashtext-2.7-py2.py3-none-any.whl size=9300 sha256=35d8913c9b81ad238a42657b56073fd7c416f764cecd9f6502891c587b29cb8a
 Stored in directory: /root/.cache/pip/wheels/49/20/47/f03dfa8a7239c54cbc44ff7389eefbf888d2c1873edaaec888
Successfully built flashtext
Installing collected packages: flashtext, blis, thinc, scipy, scikit-learn, scattertext
 Attempting uninstall: blis
 Found existing installation: blis 1.3.0
 Uninstalling blis-1.3.0:
 Successfully uninstalled blis-1.3.0
 Attempting uninstall: thinc
 Found existing installation: thinc 8.3.6
 Uninstalling thinc-8.3.6:
 Successfully uninstalled thinc-8.3.6
 Attempting uninstall: scipy
 Found existing installation: scipy 1.15.3
 Uninstalling scipy-1.15.3:
 Successfully uninstalled scipy-1.15.3
 Attempting uninstall: scikit-learn
 Found existing installation: scikit-learn 1.2.2
 Uninstalling scikit-learn-1.2.2:
 Successfully uninstalled scikit-learn-1.2.2
ERROR: pip's dependency resolver does not currently take into account all the packages that are installed. This behaviour is the source of the following dependency conflicts.
category-encoders 2.7.0 requires scikit-learn<1.6.0,>=1.0.0, but you have scikit-learn 1.7.2 which is incompatible.
cesium 0.12.4 requires numpy<3.0,>=2.0, but you have numpy 1.26.4 which is incompatible.
tsfresh 0.21.0 requires scipy>=1.14.0; python_version >= "3.10", but you have scipy 1.13.1 which is incompatible.
dopamine-rl 4.1.2 requires gymnasium>=1.0.0, but you have gymnasium 0.29.0 which is incompatible.
sklearn-compat 0.1.3 requires scikit-learn<1.7,>=1.2, but you have scikit-learn 1.7.2 which is incompatible.
plotnine 0.14.5 requires matplotlib>=3.8.0, but you have matplotlib 3.7.2 which is incompatible.
Successfully installed blis-1.2.1 flashtext-2.7 scattertext-0.2.2 scikit-learn-1.7.2 scipy-1.13.1 thinc-8.3.4
Requirement already satisfied: nltk in /usr/local/lib/python3.11/dist-packages (3.9.1)
Requirement already satisfied: click in /usr/local/lib/python3.11/dist-packages (from nltk) (8.3.0)
Requirement already satisfied: joblib in /usr/local/lib/python3.11/dist-packages (from nltk) (1.5.2)
Requirement already satisfied: regex>=2021.8.3 in /usr/local/lib/python3.11/dist-packages (from nltk) (2025.9.18)
Requirement already satisfied: tqdm in /usr/local/lib/python3.11/dist-packages (from nltk) (4.67.1)
Requirement already satisfied: kagglehub in /usr/local/lib/python3.11/dist-packages (0.3.13)
Requirement already satisfied: packaging in /usr/local/lib/python3.11/dist-packages (from kagglehub) (25.0)
Requirement already satisfied: pyyaml in /usr/local/lib/python3.11/dist-packages (from kagglehub) (6.0.3)
Requirement already satisfied: requests in /usr/local/lib/python3.11/dist-packages (from kagglehub) (2.32.5)
Requirement already satisfied: tqdm in /usr/local/lib/python3.11/dist-packages (from kagglehub) (4.67.1)
Requirement already satisfied: charset_normalizer<4,>=2 in /usr/local/lib/python3.11/dist-packages (from requests->kagglehub) (3.4.3)
Requirement already satisfied: idna<4,>=2.5 in /usr/local/lib/python3.11/dist-packages (from requests->kagglehub) (3.10)
Requirement already satisfied: urllib3<3,>=1.21.1 in /usr/local/lib/python3.11/dist-packages (from requests->kagglehub) (2.5.0)
Requirement already satisfied: certifi>=2017.4.17 in /usr/local/lib/python3.11/dist-packages (from requests->kagglehub) (2025.8.3)

!pip install --upgrade numpy
!pip install scipy==<desired_version>

import tweepy
from textblob import TextBlob
from wordcloud import WordCloud
import pandas as pd
import numpy as np
import re
import matplotlib.pyplot as plt
plt.style.use('fivethirtyeight')
# Import Data Preprocessing and Wrangling libraries
import re
from tqdm.notebook import tqdm
import pandas as pd
import numpy as np
from datetime import datetime

# Import NLP Libraries
import nltk
from spellchecker import SpellChecker
from nltk.sentiment.vader import SentimentIntensityAnalyzer as SIA

# Import Visualization Libraries
import plotly.offline as pyo
import plotly.express as px
import plotly.graph_objects as go
import matplotlib.pyplot as plt
from plotly.subplots import make_subplots
import seaborn as sns
import scattertext as st
from IPython.display import IFrame
from wordcloud import WordCloud, ImageColorGenerator
import matplotlib.pyplot as plt
from nltk.corpus import stopwords
import random

# Downloading periphrals
nltk.download('vader_lexicon')
nltk.download('stopwords')

# Remove distarcting warning
import warnings
warnings.filterwarnings('ignore')

2025-10-26 04:20:13.477568: E external/local_xla/xla/stream_executor/cuda/cuda_fft.cc:477] Unable to register cuFFT factory: Attempting to register factory for plugin cuFFT when one has already been registered
WARNING: All log messages before absl::InitializeLog() is called are written to STDERR
E0000 00:00:1761452413.660412 37 cuda_dnn.cc:8310] Unable to register cuDNN factory: Attempting to register factory for plugin cuDNN when one has already been registered
E0000 00:00:1761452413.716762 37 cuda_blas.cc:1418] Unable to register cuBLAS factory: Attempting to register factory for plugin cuBLAS when one has already been registered
[nltk_data] Downloading package vader_lexicon to
[nltk_data] /usr/share/nltk_data...
[nltk_data] Package vader_lexicon is already up-to-date!
[nltk_data] Downloading package stopwords to /usr/share/nltk_data...
[nltk_data] Package stopwords is already up-to-date!

df=pd.read_csv('/kaggle/input/review-2025/1 deepseek reviews 2025.1.20-2025.10.25.csv')
df=df.astype(str)

df.head(5)

tweet
0 this aap is tooo slow
1 very good
2 Good
3 it is a muslim robot that tries to answer reli...
4 over the course of time Dumb ChatGPT improved ...

df.tail(5)

tweet
39303 Nothing can be said but the best
39304 Good model. Barebones/rushed app, Fix UI Asap🤝
39305 目前的使用体验非常好，app简洁实用，没有赘余的功能和设计，这是非常棒的一点，比目前来自中国...
39306 This app is amazing! It’s super helpful, easy ...
39307 Could you add "Dark mode" PLZ

import pandas as pd
import re
from textblob import TextBlob

# Assign appropriate column names to the dataframe
df.columns = ['tweet']

# Create a copy of the dataframe
data = df.copy()
data['clean_Tweet'] = df['tweet']

# Standard tweet preprocessing
data['clean_Tweet'] = data['clean_Tweet'].str.lower()
# Remove Twitter handles
data['clean_Tweet'] = data['clean_Tweet'].apply(lambda x: re.sub('@[^\s]+', '', x))
# Remove hashtags
data['clean_Tweet'] = data['clean_Tweet'].apply(lambda x: re.sub(r'\B#\S+', '', x))
# Remove URLs
data['clean_Tweet'] = data['clean_Tweet'].apply(lambda x: re.sub(r"http\S+", "", x))
# Remove all special characters
data['clean_Tweet'] = data['clean_Tweet'].apply(lambda x: ' '.join(re.findall(r'\w+', x)))
# Remove all single characters
data['clean_Tweet'] = data['clean_Tweet'].apply(lambda x: re.sub(r'\s+[a-zA-Z]\s+', '', x))
# Substituting multiple spaces with a single space
data['clean_Tweet'] = data['clean_Tweet'].apply(lambda x: re.sub(r'\s+', ' ', x, flags=re.I))

# Define the function to get subjectivity
def getSubjectivity(text):
 return TextBlob(text).sentiment.subjectivity

# Define the function to get polarity
def getPolarity(text):
 return TextBlob(text).sentiment.polarity

# Create two new columns for subjectivity and polarity
data['Subjectivity'] = data['clean_Tweet'].apply(getSubjectivity)
data['Polarity'] = data['clean_Tweet'].apply(getPolarity)

# Show the new dataframe with the new columns
data.head()

tweet \
0 this aap is tooo slow
1 very good
2 Good
3 it is a muslim robot that tries to answer reli...
4 over the course of time Dumb ChatGPT improved ...

 clean_Tweet Subjectivity Polarity
0 this aap is tooo slow 0.40 -0.300
1 very good 0.78 0.910
2 good 0.60 0.700
3 it ismuslim robot that tries to answer religio... 0.40 -0.100
4 over the course of time dumb chatgpt improvedl... 0.50 -0.375

# Load the data

# Create a function to get the subjectivity

import matplotlib.pyplot as plt
from wordcloud import WordCloud

# Get all words from the 'clean_Tweet' column
allWords = ' '.join(data['clean_Tweet'])

# Generate word cloud
wordCloud = WordCloud()

# Process text and calculate word frequencies
wordFrequencies = wordCloud.process_text(allWords)

# Sort word frequencies in descending order
sortedWordFrequencies = sorted(wordFrequencies.items(), key=lambda x: x[1], reverse=True)

# Display top 30 words
top30Words = sortedWordFrequencies[:30]
for word, frequency in top30Words:
 print(f"{word}: {frequency}")

# Generate word cloud from word frequencies
wordCloud = WordCloud(width=800, height=400, background_color='white').generate_from_frequencies(wordFrequencies)

# Display the word cloud
plt.figure(figsize=(10, 5))
plt.imshow(wordCloud, interpolation='bilinear')
plt.axis('off')

# Save the picture with tight bounding box
plt.savefig('word_cloud.png', bbox_inches='tight')
plt.show()

app: 4685
good: 3911
chatgpt: 2542
deepseek: 2507
better: 2129
server: 1884
best: 1854
answer: 1544
time: 1451
ai: 1442
best ai: 1307
use: 1305
chat gpt: 1201
great: 1185
amazing: 1104
free: 1050
one: 1005
nice: 976
busy: 976
love: 970
question: 952
thank: 903
excellent: 839
work: 833
give: 829
problem: 815
slow: 803
response: 796
much: 756
need: 754


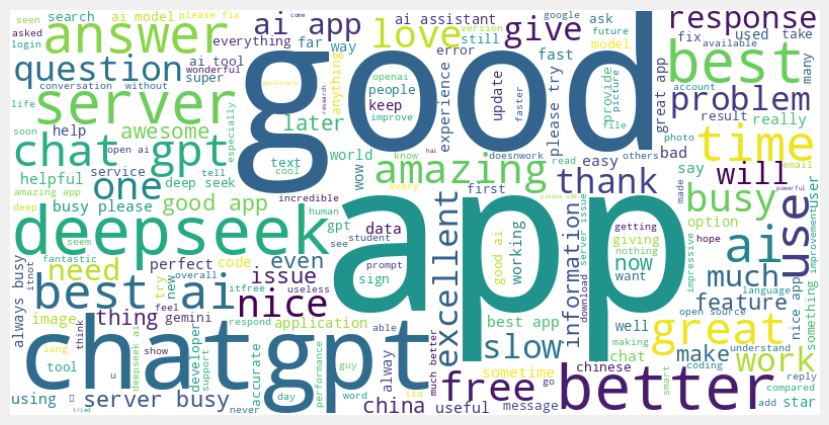


# Create a function to compute the negative, neutral and positive analysis
def getAnalysis(score):
 if score < 0:
 return 'Negative'
 elif score == 0:
 return 'Neutral'
 else:
 return 'Positive'

# Apply the function to the Polarity column
data['Analysis'] = data['Polarity'].apply(getAnalysis)

# Show the DataFrame
data.tail()

tweet \
39303 Nothing can be said but the best
39304 Good model. Barebones/rushed app, Fix UI Asap🤝
39305 目前的使用体验非常好，app简洁实用，没有赘余的功能和设计，这是非常棒的一点，比目前来自中国...
39306 This app is amazing! It’s super helpful, easy ...
39307 Could you add "Dark mode" PLZ

 clean_Tweet Subjectivity \
39303 nothing can be said but the best 0.300000
39304 good model barebones rushed app fix ui asap 0.600000
39305 目前的使用体验非常好 app简洁实用 没有赘余的功能和设计 这是非常棒的一点 比目前来自中国... 0.000000
39306 this app is amazing itsuper helpful easy to us... 0.515119
39307 could you add dark mode plz 0.400000

 Polarity Analysis
39303 1.000000 Positive
39304 0.700000 Positive
39305 0.000000 Neutral
39306 0.364762 Positive
39307 -0.150000 Negative

import matplotlib.pyplot as plt

# Plot polarity
plt.figure(figsize=(8, 6))
plt.scatter(data['Polarity'], data['Subjectivity'], color='blue', alpha=0.5)
plt.title('Sentiment Analysis')
plt.xlabel('Polarity')
plt.ylabel('Subjectivity')
plt.savefig('sentiment_analysis.png', dpi=300, bbox_inches='tight')
plt.show()


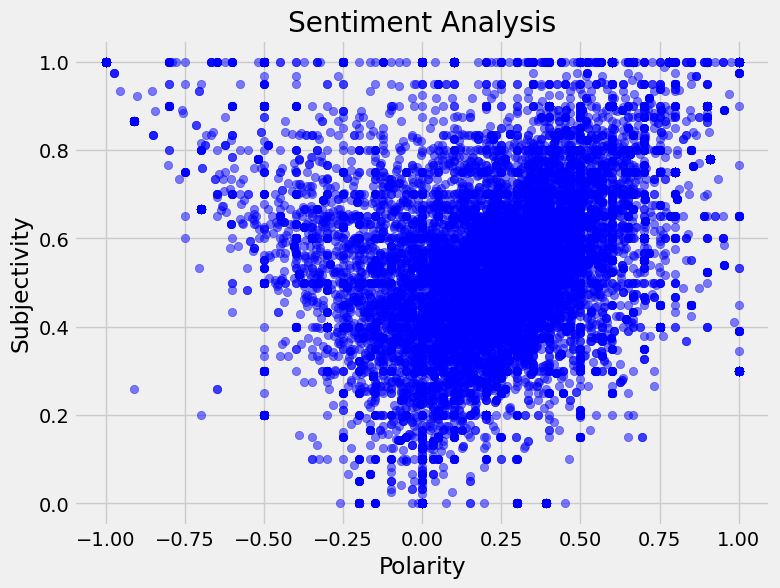


# Get the percentage of positive tweets
ptweets=data[data.Analysis=='Positive']
ptweets=ptweets['clean_Tweet']

round((ptweets.shape[0]/df.shape[0])*100,1)

68.1

# Get the percentage of negative tweets
ptweets=data[data.Analysis=='Negative']
ptweets=ptweets['clean_Tweet']

round((ptweets.shape[0]/df.shape[0])*100,1)

11.0

# Get the percentage of neutral tweets
ptweets=data[data.Analysis=='Neutral']
ptweets=ptweets['clean_Tweet']

round((ptweets.shape[0]/df.shape[0])*100,1)

20.9

import matplotlib.pyplot as plt

# Show the value counts
value_counts = data['Analysis'].value_counts()

# Plot and visualize the counts
plt.figure(figsize=(10, 6), facecolor='white') # Set the figure background to white
plt.title('Sentiment Analysis', color='black')
plt.xlabel('Sentiment', color='black')
plt.ylabel('Counts', color='black')
ax = value_counts.plot(kind='bar')

# Set the background color of the plot area to white
ax.set_facecolor('white')

# Save the plot as an image
plt.savefig('sentiment_analysis_result.png', bbox_inches='tight', facecolor='white') # Save with white background
plt.show()


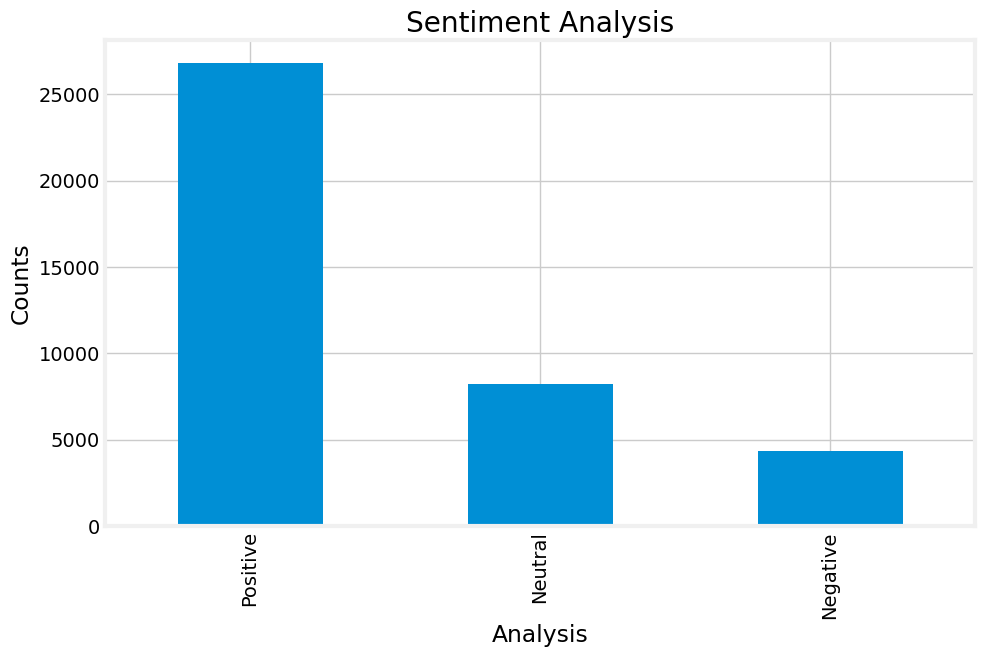


import matplotlib.pyplot as plt

# Define the function to compute sentiment analysis
def get_analysis(score):
 if score < 0:
 return 'Negative'
 elif score == 0:
 return 'Neutral'
 else:
 return 'Positive'

# Apply the function to the Polarity column
data['Analysis'] = data['Polarity'].apply(get_analysis)

# Define a harmonious color palette using complementary colors
color_map = {'Positive': '#2E8B57', 'Neutral': '#FFA500', 'Negative': '#8B4513'} # Sea Green, Orange, Saddle Brown
scatter_colors = data['Analysis'].map(color_map)

# Use the same color palette for the bar chart
bar_colors = [color_map['Positive'], color_map['Neutral'], color_map['Negative']]

# Create subplots
fig, ax = plt.subplots(1, 2, figsize=(14, 6), facecolor='white')

# Scatter plot for Polarity vs. Subjectivity (left)
ax[0].scatter(data['Polarity'], data['Subjectivity'], c=scatter_colors, alpha=0.7, edgecolors='black')
ax[0].set_title('Polarity vs Subjectivity', color='black', fontsize=14, fontweight='bold')
ax[0].set_xlabel('Polarity', color='black')
ax[0].set_ylabel('Subjectivity', color='black')
ax[0].set_facecolor('white')

# Show the value counts for sentiment analysis
value_counts = data['Analysis'].value_counts()

# Bar plot for Sentiment Analysis counts (right)
value_counts = value_counts.reindex(['Positive', 'Neutral', 'Negative']) # Maintain consistent order
value_counts.plot(kind='bar', ax=ax[1], color=bar_colors, alpha=0.7, edgecolor='black')
ax[1].set_title('Sentiment Distribution', color='black', fontsize=14, fontweight='bold')
ax[1].set_xlabel('Sentiment', color='black')
ax[1].set_ylabel('Counts', color='black')
ax[1].set_facecolor('white')

# Set font sizes and styles for both subplots
for axis in ax:
 axis.title.set_fontsize(14)
 axis.title.set_fontweight('bold')
 axis.xaxis.label.set_fontsize(12)
 axis.yaxis.label.set_fontsize(12)

# Adjust layout to avoid overlap
plt.tight_layout()

# Save the combined plot as an image
plt.savefig('combined_sentiment_plot_journal_colors.png', dpi=300, bbox_inches='tight', facecolor='white')

# Show the plot
plt.show()


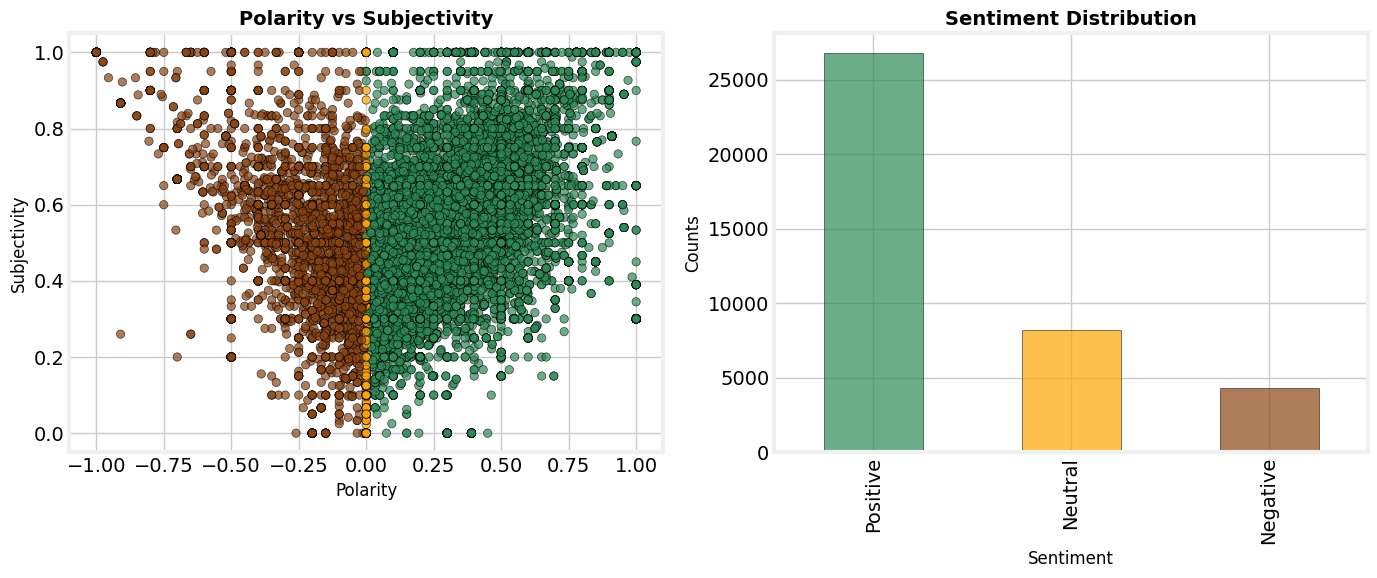

Supplement: Supplementary file 1 [file Table_1.docx]
