# Supplementary material for: Psychological determinants of GenAI adoption for foreign language education: an extended UTAUT2 model and sentiment analysis approach
Source: Front Psychol. 2026 Jan 9;16:1622926. doi: 10.3389/fpsyg.2025.1622926 (PMC12827767; doi:10.3389/fpsyg.2025.1622926)
Supplement: Supplementary file 2 [file Table_2.docx]

# **Google Play Scraper**

### Introduction

In this notebook, I’m trying to scrape Google Play reviews using Kaggle. The process is quite simple and uses the google-play-scraper library, which allows us to extract app reviews easily. For this example, I’m focusing on reviews for the Duolingo app.

### Installing Libraries

First, I installed the necessary library, google-play-scraper, which is designed for fetching data from the Google Play Store.

!pip install google-play-scraper

### Importing Libraries

After the installation, I imported the required libraries for data scraping and manipulation. This includes google-play-scraper for fetching reviews and pandas for working with the resulting dataset.

from google_play_scraper import reviews, Sort
import pandas as pd

!pip install google-play-scraper

from google_play_scraper import reviews, Sort

print('All set')

Collecting google-play-scraper
 Downloading google_play_scraper-1.2.7-py3-none-any.whl.metadata (50 kB)
━━━━━━━━━━━━━━━━━━━━━━━━━━━━━━━━━━━━━━━━ 50.2/50.2 kB 1.8 MB/s eta 0:00:00

### Scraping the Reviews

I used the reviews function to scrape the most recent reviews for the Duolingo app from Google Play. The parameters included:

- **'com.duolingo'**: the app's package name.
- **lang='en'**: reviews in English.
- **country='us'**: reviews from the U.S. store.
- **sort=Sort.NEWEST**: sorting the reviews by newest first.
- **count=100**: limiting the result to 100 reviews.

The code snippet for this step is as follows:

result, continuation_token = reviews(
 'com.duolingo',
 lang='en',
 country='us',
 sort=Sort.NEWEST,
 count=100
)

### Creating a DataFrame

I converted the scraped results into a DataFrame for better organization and easier manipulation.

df = pd.DataFrame(result)
df.head()

At this stage, the DataFrame contains various columns, including review content, score, thumbs-up count, review date, and more.

from datetime import datetime
import pandas as pd

# Initialize variables
all_reviews = []
continuation_token = None
start_date = datetime(2025, 1, 20)
end_date = datetime(2025, 10, 25)

print("Starting review scraping...")

while True:
 try:
 # Fetch reviews in batches
 result, continuation_token = reviews(
 'com.deepseek.chat',
 lang='eng',
 country='us',
 sort=Sort.NEWEST,
 count=100, # Batch size
 continuation_token=continuation_token
 )

 if not result:
 break

 # Convert to DataFrame for easier filtering
 batch_df = pd.DataFrame(result)

 # Convert timestamp column
 if 'at' in batch_df.columns:
 batch_df['at'] = pd.to_datetime(batch_df['at'])
 # Filter by date range
 batch_df = batch_df[(batch_df['at'] >= start_date) & (batch_df['at'] <= end_date)]
 elif 'timestamp' in batch_df.columns:
 batch_df['timestamp'] = pd.to_datetime(batch_df['timestamp'])
 batch_df = batch_df[(batch_df['timestamp'] >= start_date) & (batch_df['timestamp'] <= end_date)]

 # Add filtered reviews to main list
 all_reviews.extend(batch_df.to_dict('records'))

 print(f"Fetched {len(batch_df)} reviews in this batch")

 # Stop if we've gone beyond our start date
 if len(batch_df) > 0:
 oldest_review_date = batch_df['at'].min() if 'at' in batch_df.columns else batch_df['timestamp'].min()
 if oldest_review_date < start_date:
 break

 if continuation_token is None:
 break

 except Exception as e:
 print(f"Error occurred: {e}")
 break

# Convert all collected reviews to DataFrame
df = pd.DataFrame(all_reviews)

print(f"Total reviews collected: {len(df)}")
print(f"Date range: {df['at'].min() if 'at' in df.columns else df['timestamp'].min()} to {df['at'].max() if 'at' in df.columns else df['timestamp'].max()}")

# Display the first few rows
df.head()

Starting review scraping...
Fetched 98 reviews in this batch
Fetched 100 reviews in this batch
Fetched 100 reviews in this batch
Fetched 100 reviews in this batch
Fetched 100 reviews in this batch
Fetched 100 reviews in this batch
Fetched 100 reviews in this batch
Fetched 100 reviews in this batch
Fetched 100 reviews in this batch
Fetched 100 reviews in this batch
Fetched 100 reviews in this batch
Fetched 100 reviews in this batch
Fetched 100 reviews in this batch
Fetched 100 reviews in this batch
Fetched 100 reviews in this batch
Fetched 100 reviews in this batch
Fetched 100 reviews in this batch
Fetched 100 reviews in this batch
Fetched 100 reviews in this batch
Fetched 100 reviews in this batch
Fetched 100 reviews in this batch
Fetched 100 reviews in this batch
Fetched 100 reviews in this batch
Fetched 100 reviews in this batch
Fetched 100 reviews in this batch
Fetched 100 reviews in this batch
Fetched 100 reviews in this batch
Fetched 100 reviews in this batch
Fetched 100 reviews in this batch
Fetched 100 reviews in this batch
Fetched 100 reviews in this batch
Fetched 100 reviews in this batch
Fetched 100 reviews in this batch
Fetched 100 reviews in this batch
Fetched 100 reviews in this batch
Fetched 100 reviews in this batch
Fetched 100 reviews in this batch
Fetched 100 reviews in this batch
Fetched 100 reviews in this batch
Fetched 100 reviews in this batch
Fetched 100 reviews in this batch
Fetched 100 reviews in this batch
Fetched 100 reviews in this batch
Fetched 100 reviews in this batch
Fetched 100 reviews in this batch
Fetched 100 reviews in this batch
Fetched 100 reviews in this batch
Fetched 100 reviews in this batch
Fetched 100 reviews in this batch
Fetched 100 reviews in this batch
Fetched 100 reviews in this batch
Fetched 100 reviews in this batch
Fetched 100 reviews in this batch
Fetched 100 reviews in this batch
Fetched 100 reviews in this batch
Fetched 100 reviews in this batch
Fetched 100 reviews in this batch
Fetched 100 reviews in this batch
Fetched 100 reviews in this batch
Fetched 100 reviews in this batch
Fetched 100 reviews in this batch
Fetched 100 reviews in this batch
Fetched 100 reviews in this batch
Fetched 100 reviews in this batch
Fetched 100 reviews in this batch
Fetched 100 reviews in this batch
Fetched 100 reviews in this batch
Fetched 100 reviews in this batch
Fetched 100 reviews in this batch
Fetched 100 reviews in this batch
Fetched 100 reviews in this batch
Fetched 100 reviews in this batch
Fetched 100 reviews in this batch
Fetched 100 reviews in this batch
Fetched 100 reviews in this batch
Fetched 100 reviews in this batch
Fetched 100 reviews in this batch
Fetched 100 reviews in this batch
Fetched 100 reviews in this batch
Fetched 100 reviews in this batch
Fetched 100 reviews in this batch
Fetched 100 reviews in this batch
Fetched 100 reviews in this batch
Fetched 100 reviews in this batch
Fetched 100 reviews in this batch
Fetched 100 reviews in this batch
Fetched 100 reviews in this batch
Fetched 100 reviews in this batch
Fetched 100 reviews in this batch
Fetched 100 reviews in this batch
Fetched 100 reviews in this batch
Fetched 100 reviews in this batch
Fetched 100 reviews in this batch
Fetched 100 reviews in this batch
Fetched 100 reviews in this batch
Fetched 100 reviews in this batch
Fetched 100 reviews in this batch
Fetched 100 reviews in this batch
Fetched 100 reviews in this batch
Fetched 100 reviews in this batch
Fetched 100 reviews in this batch
Fetched 100 reviews in this batch
Fetched 100 reviews in this batch
Fetched 100 reviews in this batch
Fetched 100 reviews in this batch
Fetched 100 reviews in this batch
Fetched 100 reviews in this batch
Fetched 100 reviews in this batch
Fetched 100 reviews in this batch
Fetched 100 reviews in this batch
Fetched 100 reviews in this batch
Fetched 100 reviews in this batch
Fetched 100 reviews in this batch
Fetched 100 reviews in this batch
Fetched 100 reviews in this batch
Fetched 100 reviews in this batch
Fetched 100 reviews in this batch
Fetched 100 reviews in this batch
Fetched 100 reviews in this batch
Fetched 100 reviews in this batch
Fetched 100 reviews in this batch
Fetched 100 reviews in this batch
Fetched 100 reviews in this batch
Fetched 100 reviews in this batch
Fetched 100 reviews in this batch
Fetched 100 reviews in this batch
Fetched 100 reviews in this batch
Fetched 100 reviews in this batch
Fetched 100 reviews in this batch
Fetched 100 reviews in this batch
Fetched 100 reviews in this batch
Fetched 100 reviews in this batch
Fetched 100 reviews in this batch
Fetched 100 reviews in this batch
Fetched 100 reviews in this batch
Fetched 100 reviews in this batch
Fetched 100 reviews in this batch
Fetched 100 reviews in this batch
Fetched 100 reviews in this batch
Fetched 100 reviews in this batch
Fetched 100 reviews in this batch
Fetched 100 reviews in this batch
Fetched 100 reviews in this batch
Fetched 100 reviews in this batch
Fetched 100 reviews in this batch
Fetched 100 reviews in this batch
Fetched 100 reviews in this batch
Fetched 100 reviews in this batch
Fetched 100 reviews in this batch
Fetched 100 reviews in this batch
Fetched 100 reviews in this batch
Fetched 100 reviews in this batch
Fetched 100 reviews in this batch
Fetched 100 reviews in this batch
Fetched 100 reviews in this batch
Fetched 100 reviews in this batch
Fetched 100 reviews in this batch
Fetched 100 reviews in this batch
Fetched 100 reviews in this batch
Fetched 100 reviews in this batch
Fetched 100 reviews in this batch
Fetched 100 reviews in this batch
Fetched 100 reviews in this batch
Fetched 100 reviews in this batch
Fetched 100 reviews in this batch
Fetched 100 reviews in this batch
Fetched 100 reviews in this batch
Fetched 100 reviews in this batch
Fetched 100 reviews in this batch
Fetched 100 reviews in this batch
Fetched 100 reviews in this batch
Fetched 100 reviews in this batch
Fetched 100 reviews in this batch
Fetched 100 reviews in this batch
Fetched 100 reviews in this batch
Fetched 100 reviews in this batch
Fetched 100 reviews in this batch
Fetched 100 reviews in this batch
Fetched 100 reviews in this batch
Fetched 100 reviews in this batch
Fetched 100 reviews in this batch
Fetched 100 reviews in this batch
Fetched 100 reviews in this batch
Fetched 100 reviews in this batch
Fetched 100 reviews in this batch
Fetched 100 reviews in this batch
Fetched 100 reviews in this batch
Fetched 100 reviews in this batch
Fetched 100 reviews in this batch
Fetched 100 reviews in this batch
Fetched 100 reviews in this batch
Fetched 100 reviews in this batch
Fetched 100 reviews in this batch
Fetched 100 reviews in this batch
Fetched 100 reviews in this batch
Fetched 100 reviews in this batch
Fetched 100 reviews in this batch
Fetched 100 reviews in this batch
Fetched 100 reviews in this batch
Fetched 100 reviews in this batch
Fetched 100 reviews in this batch
Fetched 100 reviews in this batch
Fetched 100 reviews in this batch
Fetched 100 reviews in this batch
Fetched 100 reviews in this batch
Fetched 100 reviews in this batch
Fetched 100 reviews in this batch
Fetched 100 reviews in this batch
Fetched 100 reviews in this batch
Fetched 100 reviews in this batch
Fetched 100 reviews in this batch
Fetched 100 reviews in this batch
Fetched 100 reviews in this batch
Fetched 100 reviews in this batch
Fetched 100 reviews in this batch
Fetched 100 reviews in this batch
Fetched 100 reviews in this batch
Fetched 100 reviews in this batch
Fetched 100 reviews in this batch
Fetched 100 reviews in this batch
Fetched 100 reviews in this batch
Fetched 100 reviews in this batch
Fetched 100 reviews in this batch
Fetched 100 reviews in this batch
Fetched 100 reviews in this batch
Fetched 100 reviews in this batch
Fetched 100 reviews in this batch
Fetched 100 reviews in this batch
Fetched 100 reviews in this batch
Fetched 100 reviews in this batch
Fetched 100 reviews in this batch
Fetched 100 reviews in this batch
Fetched 100 reviews in this batch
Fetched 100 reviews in this batch
Fetched 100 reviews in this batch
Fetched 100 reviews in this batch
Fetched 100 reviews in this batch
Fetched 100 reviews in this batch
Fetched 100 reviews in this batch
Fetched 100 reviews in this batch
Fetched 100 reviews in this batch
Fetched 100 reviews in this batch
Fetched 100 reviews in this batch
Fetched 100 reviews in this batch
Fetched 100 reviews in this batch
Fetched 100 reviews in this batch
Fetched 100 reviews in this batch
Fetched 100 reviews in this batch
Fetched 100 reviews in this batch
Fetched 100 reviews in this batch
Fetched 100 reviews in this batch
Fetched 100 reviews in this batch
Fetched 100 reviews in this batch
Fetched 100 reviews in this batch
Fetched 100 reviews in this batch
Fetched 100 reviews in this batch
Fetched 100 reviews in this batch
Fetched 100 reviews in this batch
Fetched 100 reviews in this batch
Fetched 100 reviews in this batch
Fetched 100 reviews in this batch
Fetched 100 reviews in this batch
Fetched 100 reviews in this batch
Fetched 100 reviews in this batch
Fetched 100 reviews in this batch
Fetched 100 reviews in this batch
Fetched 100 reviews in this batch
Fetched 100 reviews in this batch
Fetched 100 reviews in this batch
Fetched 100 reviews in this batch
Fetched 100 reviews in this batch
Fetched 100 reviews in this batch
Fetched 100 reviews in this batch
Fetched 100 reviews in this batch
Fetched 100 reviews in this batch
Fetched 100 reviews in this batch
Fetched 100 reviews in this batch
Fetched 100 reviews in this batch
Fetched 100 reviews in this batch
Fetched 100 reviews in this batch
Fetched 100 reviews in this batch
Fetched 100 reviews in this batch
Fetched 100 reviews in this batch
Fetched 100 reviews in this batch
Fetched 100 reviews in this batch
Fetched 100 reviews in this batch
Fetched 100 reviews in this batch
Fetched 100 reviews in this batch
Fetched 100 reviews in this batch
Fetched 100 reviews in this batch
Fetched 100 reviews in this batch
Fetched 100 reviews in this batch
Fetched 100 reviews in this batch
Fetched 100 reviews in this batch
Fetched 100 reviews in this batch
Fetched 100 reviews in this batch
Fetched 100 reviews in this batch
Fetched 100 reviews in this batch
Fetched 100 reviews in this batch
Fetched 100 reviews in this batch
Fetched 100 reviews in this batch
Fetched 100 reviews in this batch
Fetched 100 reviews in this batch
Fetched 100 reviews in this batch
Fetched 100 reviews in this batch
Fetched 100 reviews in this batch
Fetched 100 reviews in this batch
Fetched 100 reviews in this batch
Fetched 100 reviews in this batch
Fetched 100 reviews in this batch
Fetched 100 reviews in this batch
Fetched 100 reviews in this batch
Fetched 100 reviews in this batch
Fetched 100 reviews in this batch
Fetched 100 reviews in this batch
Fetched 100 reviews in this batch
Fetched 100 reviews in this batch
Fetched 100 reviews in this batch
Fetched 100 reviews in this batch
Fetched 100 reviews in this batch
Fetched 100 reviews in this batch
Fetched 100 reviews in this batch
Fetched 100 reviews in this batch
Fetched 100 reviews in this batch
Fetched 100 reviews in this batch
Fetched 100 reviews in this batch
Fetched 100 reviews in this batch
Fetched 100 reviews in this batch
Fetched 100 reviews in this batch
Fetched 100 reviews in this batch
Fetched 100 reviews in this batch
Fetched 100 reviews in this batch
Fetched 100 reviews in this batch
Fetched 100 reviews in this batch
Fetched 100 reviews in this batch
Fetched 100 reviews in this batch
Fetched 100 reviews in this batch
Fetched 100 reviews in this batch
Fetched 100 reviews in this batch
Fetched 100 reviews in this batch
Fetched 100 reviews in this batch
Fetched 100 reviews in this batch
Fetched 100 reviews in this batch
Fetched 100 reviews in this batch
Fetched 100 reviews in this batch
Fetched 100 reviews in this batch
Fetched 100 reviews in this batch
Fetched 100 reviews in this batch
Fetched 100 reviews in this batch
Fetched 100 reviews in this batch
Fetched 100 reviews in this batch
Fetched 100 reviews in this batch
Fetched 100 reviews in this batch
Fetched 100 reviews in this batch
Fetched 100 reviews in this batch
Fetched 100 reviews in this batch
Fetched 100 reviews in this batch
Fetched 100 reviews in this batch
Fetched 100 reviews in this batch
Fetched 100 reviews in this batch
Fetched 100 reviews in this batch
Fetched 100 reviews in this batch
Fetched 100 reviews in this batch
Fetched 100 reviews in this batch
Fetched 100 reviews in this batch
Fetched 100 reviews in this batch
Fetched 100 reviews in this batch
Fetched 100 reviews in this batch
Fetched 100 reviews in this batch
Fetched 100 reviews in this batch
Fetched 100 reviews in this batch
Fetched 100 reviews in this batch
Fetched 100 reviews in this batch
Fetched 100 reviews in this batch
Fetched 100 reviews in this batch
Fetched 100 reviews in this batch
Fetched 100 reviews in this batch
Fetched 100 reviews in this batch
Fetched 100 reviews in this batch
Fetched 100 reviews in this batch
Fetched 100 reviews in this batch
Fetched 100 reviews in this batch
Fetched 100 reviews in this batch
Fetched 100 reviews in this batch
Fetched 100 reviews in this batch
Fetched 100 reviews in this batch
Fetched 100 reviews in this batch
Fetched 100 reviews in this batch
Fetched 100 reviews in this batch
Fetched 100 reviews in this batch
Fetched 100 reviews in this batch
Fetched 100 reviews in this batch
Fetched 100 reviews in this batch
Fetched 12 reviews in this batch
Fetched 0 reviews in this batch
Fetched 0 reviews in this batch
Total reviews collected: 39310
Date range: 2025-01-20 01:15:29 to 2025-10-24 23:54:17

reviewId userName \
0 c4072513-61b3-450a-879e-f0223107bbed Mahesh prasad Gupta
1 043a19bb-b713-46b9-9332-319446ade712 Alin Walin
2 d995f6d8-c8b6-4610-a083-5dd9188b26b5 Sepehr Bashirzadeh
3 0094864f-7f1c-4b08-96e4-52efbcf16878 Zaid
4 9d6fa765-6556-41e6-af24-7082ddef2b9b Prince David

 userImage \
0 https://play-lh.googleusercontent.com/a/ACg8oc...
1 https://play-lh.googleusercontent.com/a-/ALV-U...
2 https://play-lh.googleusercontent.com/a/ACg8oc...
3 https://play-lh.googleusercontent.com/a/ACg8oc...
4 https://play-lh.googleusercontent.com/a-/ALV-U...

 content score thumbsUpCount \
0 this aap is tooo slow 1 0
1 very good 5 0
2 Good 5 0
3 it is a muslim robot that tries to answer reli... 1 0
4 over the course of time Dumb ChatGPT improved ... 3 0

 reviewCreatedVersion at replyContent repliedAt appVersion
0 None 2025-10-24 23:54:17 None NaT None
1 1.4.3 2025-10-24 22:11:12 None NaT 1.4.3
2 1.4.3 2025-10-24 21:31:32 None NaT 1.4.3
3 1.4.3 2025-10-24 20:29:25 None NaT 1.4.3
4 1.4.3 2025-10-24 20:22:04 None NaT 1.4.3

### Saving the Data

To save the cleaned reviews for future use or analysis, I exported the DataFrame into a CSV file.

df.to_csv('google_play_reviews.csv', index=False)

This CSV file can now be downloaded and shared for further analysis.

# Save to CSV
df.to_csv('google_play_reviews.csv', index=False)

# Check the saved file
print("File saved successfully!")

File saved successfully!

# Download the file (if running locally or need it for later)
from IPython.display import FileLink
FileLink('google_play_reviews.csv')

/kaggle/working/google_play_reviews.csv

### Conclusion

This notebook demonstrates a quick and efficient way to scrape reviews from Google Play and organize them in a structured format using Kaggle. The dataset is ready to be used for tasks such as text analysis, sentiment analysis, or any other exploratory work.
