# Supplementary material for: Psychological determinants of GenAI adoption for foreign language education: an extended UTAUT2 model and sentiment analysis approach
Source: Front Psychol. 2026 Jan 9;16:1622926. doi: 10.3389/fpsyg.2025.1622926 (PMC12827767; doi:10.3389/fpsyg.2025.1622926)
Supplement: Supplementary file 3 [file Table_3.docx]

**DeepSeek/深度求索**

Dear Participant, / 尊敬的受访者，

DeepSeek (https://www.deepseek.com) is an advanced AI-powered tool designed to enhance educational and professional tasks by leveraging artificial intelligence and natural language processing. We are conducting a study to explore the factors influencing people's intention to adopt DeepSeek AI for foreign language education. Your participation in this survey is highly valued. It will contribute to a deeper understanding of user engagement with AI-assisted tools in education and professional settings. All responses will be kept confidential and used solely for academic purposes. Thank you for your valuable time and input.

DeepSeek (https://www.deepseek.com) 是一款先进的AI工具，旨在通过人工智能和自然语言处理技术来增强教育和专业任务。我们正在进行一项研究，探讨影响用户采用DeepSeek AI进行外语教育的因素。您的参与对我们具有重要价值，将有助于深入了解用户与AI辅助工具的互动。所有回答将严格保密，仅用于学术研究。感谢您宝贵的时间和意见。

This survey will take approximately 3 minutes. All data will remain confidential, used solely for academic research, and may be published in accordance with ethical guidelines. No personal information will be disclosed. /本次调查耗时约3分钟。所有数据严格保密，仅用于学术研究，并可能遵循伦理准则发表。不会泄露任何个人信息。

Voluntary Participation Notice /自愿参与声明

I voluntarily agree to participate and may withdraw at any time without penalty. Data will be retained for research unless deletion is requested. /我自愿参与本调查，可随时无责退出。除非明确要求删除，数据将保留用于研究。

Sincerely, / 此致，

Research Team / 研究团队

Demographic Information / 人口统计信息

**1. Age / 年龄**

[单选题]

○ Under 20 / 20岁以下

○ 20-29 / 20-29岁

○ 30-39 / 30-39岁

○ 40-49 / 40-49岁

○ Above 50 / 50岁以上

**2. Gender / 性别**

[单选题]

○ Male / 男

○ Female / 女

**3. Education Background / 学历**

[单选题]

○ Undergraduate / 本科

○ Master's / 硕士

○ Ph.D. / Doctorate / 博士/博士后

○ Others (Diploma, Certificate courses, etc.) / 其他（文凭、证书课程等）

**4. Experience in Using AI Tools / 使用AI工具的经验**

[单选题]

○ Less than 1 year / 少于1年

○ 1-3 years / 1-3年

○ 4-6 years / 4-6年

○ More than 6 years / 超过6年

**5. Have you used DeepSeek AI before? / 您之前使用过DeepSeek AI吗？**

[单选题]

○ Yes / 是

○ No / 否

**AI Anxiety (AA) /** 人工智能焦虑 **(AA)**

6. AA1: I'm uneasy about AI, not a human teacher, assessing my foreign language speaking and writing. / 一想到由AI而非真人老师来点评我的外语口语和写作，我便感到不安。

[单选题]

| ○Not integral /不重要 | ○Somewhat integral /有点重要 | ○Moderately integral /中等重要 | ○Very integral /非常重要 | ○Extremely integral /极其重要 |
| --- | --- | --- | --- | --- |

7. AA2: Relying on DeepSeek for language learning may make my skills too tech-dependent and superficial. / 担心依赖DeepSeek学语言，会让我的能力过度依赖技术，理解流于表面。

[单选题]

| ○Not integral /不重要 | ○Somewhat integral /有点重要 | ○Moderately integral /中等重要 | ○Very integral /非常重要 | ○Extremely integral /极其重要 |
| --- | --- | --- | --- | --- |

8. AA3: Learning with a highly intelligent AI makes me feel a loss of control over my education. / 与高智能AI互动学习，让我感觉失去了对学习过程的掌控。

[单选题]

| ○Not integral /不重要 | ○Somewhat integral /有点重要 | ○Moderately integral /中等重要 | ○Very integral /非常重要 | ○Extremely integral /极其重要 |
| --- | --- | --- | --- | --- |

9. AA4: I'm concerned that AI like DeepSeek may miss language nuances, cultural context, or emotional depth. / 像DeepSeek这样的AI，或许无法真正把握语言的微妙之处、文化背景与情感内涵。

[单选题]

| ○Not integral /不重要 | ○Somewhat integral /有点重要 | ○Moderately integral /中等重要 | ○Very integral /非常重要 | ○Extremely integral /极其重要 |
| --- | --- | --- | --- | --- |

**Performance Expectancy (PE) / 绩效期望 (PE)**

10. PE1: Using DeepSeek AI improves the quality of my work. / 使用DeepSeek AI提高了我工作的质量。

[单选题]

| ○Not integral /不重要 | ○Somewhat integral /有点重要 | ○Moderately integral /中等重要 | ○Very integral /非常重要 | ○Extremely integral /极其重要 |
| --- | --- | --- | --- | --- |

11. PE2: Using DeepSeek AI increases my productivity. / 使用DeepSeek AI提高了我的生产力。

[单选题]

| ○Not integral /不重要 | ○Somewhat integral /有点重要 | ○Moderately integral /中等重要 | ○Very integral /非常重要 | ○Extremely integral /极其重要 |
| --- | --- | --- | --- | --- |

12. PE3: Using DeepSeek AI enhances my ability to handle complex tasks. / 使用DeepSeek AI增强了我处理复杂任务的能力。

[单选题]

| ○Not integral /不重要 | ○Somewhat integral /有点重要 | ○Moderately integral /中等重要 | ○Very integral /非常重要 | ○Extremely integral /极其重要 |
| --- | --- | --- | --- | --- |

**Effort Expectancy (EE) / 努力期望 (EE)**

13. EE1: Learning to use DeepSeek AI is easy for me. / 学习使用DeepSeek AI对我来说很容易。

[单选题]

| ○Not integral /不重要 | ○Somewhat integral /有点重要 | ○Moderately integral /中等重要 | ○Very integral /非常重要 | ○Extremely integral /极其重要 |
| --- | --- | --- | --- | --- |

14. EE2: I expect minimal effort in using DeepSeek AI. / 我预计使用DeepSeek AI需要的努力很小。

[单选题]

| ○Not integral /不重要 | ○Somewhat integral /有点重要 | ○Moderately integral /中等重要 | ○Very integral /非常重要 | ○Extremely integral /极其重要 |
| --- | --- | --- | --- | --- |

15. EE3: I can quickly become proficient in using DeepSeek AI. / 我能迅速掌握使用DeepSeek AI的技巧。

[单选题]

| ○Not integral /不重要 | ○Somewhat integral /有点重要 | ○Moderately integral /中等重要 | ○Very integral /非常重要 | ○Extremely integral /极其重要 |
| --- | --- | --- | --- | --- |

**Social Influence (SI) / 社会影响 (SI)**

16. SI1: People whose opinions I value recommend using DeepSeek AI. / 我重视的人推荐使用DeepSeek AI。

[单选题]

| ○Not integral /不重要 | ○Somewhat integral /有点重要 | ○Moderately integral /中等重要 | ○Very integral /非常重要 | ○Extremely integral /极其重要 |
| --- | --- | --- | --- | --- |

17. SI2: In my social or professional circles, using AI tools is encouraged. / 在我的社交或职业圈子中，鼓励使用AI工具。

[单选题]

| ○Not integral /不重要 | ○Somewhat integral /有点重要 | ○Moderately integral /中等重要 | ○Very integral /非常重要 | ○Extremely integral /极其重要 |
| --- | --- | --- | --- | --- |

18. SI3: I am influenced by the positive experiences of others who use DeepSeek AI. / 我受到了其他人使用DeepSeek AI的正面经验的影响。

[单选题]

| ○Not integral /不重要 | ○Somewhat integral /有点重要 | ○Moderately integral /中等重要 | ○Very integral /非常重要 | ○Extremely integral /极其重要 |
| --- | --- | --- | --- | --- |

**Facilitating Conditions (FC) / 便利条件 (FC)**

19. FC1: I have access to the necessary devices and internet connection to use DeepSeek AI. / 我有使用DeepSeek AI所需的设备和网络连接。

[单选题]

| ○Not integral /不重要 | ○Somewhat integral /有点重要 | ○Moderately integral /中等重要 | ○Very integral /非常重要 | ○Extremely integral /极其重要 |
| --- | --- | --- | --- | --- |

20. FC2: I am confident in learning and using new technologies. / 我对学习和使用新技术充满信心。

[单选题]

| ○Not integral /不重要 | ○Somewhat integral /有点重要 | ○Moderately integral /中等重要 | ○Very integral /非常重要 | ○Extremely integral /极其重要 |
| --- | --- | --- | --- | --- |

21. FC3: I can easily find support or assistance using DeepSeek AI if needed. / 如果需要，我可以轻松找到使用DeepSeek AI的支持或帮助。

[单选题]

| ○Not integral /不重要 | ○Somewhat integral /有点重要 | ○Moderately integral /中等重要 | ○Very integral /非常重要 | ○Extremely integral /极其重要 |
| --- | --- | --- | --- | --- |

**Hedonic Motivation (HM) / 享乐动机 (HM)**

22. HM1: I find using AI tools enjoyable. / 我觉得使用AI工具很有趣。

[单选题]

| ○Not integral /不重要 | ○Somewhat integral /有点重要 | ○Moderately integral /中等重要 | ○Very integral /非常重要 | ○Extremely integral /极其重要 |
| --- | --- | --- | --- | --- |

23. HM2: Using DeepSeek AI makes tasks more enjoyable. / 使用DeepSeek AI让任务变得更有趣。

[单选题]

| ○Not integral /不重要 | ○Somewhat integral /有点重要 | ○Moderately integral /中等重要 | ○Very integral /非常重要 | ○Extremely integral /极其重要 |
| --- | --- | --- | --- | --- |

24. HM3: I believe using DeepSeek AI will provide a sense of accomplishment. / 我相信使用DeepSeek AI会带来成就感。

[单选题]

| ○Not integral /不重要 | ○Somewhat integral /有点重要 | ○Moderately integral /中等重要 | ○Very integral /非常重要 | ○Extremely integral /极其重要 |
| --- | --- | --- | --- | --- |

**Price Value (PV) / 价格价值 (PV)**

25. PV1: I consider DeepSeek AI's pricing reasonable. / 我认为DeepSeek AI的定价合理。

[单选题]

| ○Not integral /不重要 | ○Somewhat integral /有点重要 | ○Moderately integral /中等重要 | ○Very integral /非常重要 | ○Extremely integral /极其重要 |
| --- | --- | --- | --- | --- |

26. PV2: The benefits of using DeepSeek AI outweigh its cost. / 使用DeepSeek AI的好处超过其成本。

[单选题]

| ○Not integral /不重要 | ○Somewhat integral /有点重要 | ○Moderately integral /中等重要 | ○Very integral /非常重要 | ○Extremely integral /极其重要 |
| --- | --- | --- | --- | --- |

27. PV3: Compared to other AI tools, DeepSeek AI offers good value for its price. / 与其他AI工具相比，DeepSeek AI提供了良好的价格价值。

[单选题]

| ○Not integral /不重要 | ○Somewhat integral /有点重要 | ○Moderately integral /中等重要 | ○Very integral /非常重要 | ○Extremely integral /极其重要 |
| --- | --- | --- | --- | --- |

**Habit (HB) / 习惯 (HB)**

28. HB1: I have used other AI tools before. / 我之前使用过其他AI工具。

[单选题]

| ○Not integral /不重要 | ○Somewhat integral /有点重要 | ○Moderately integral /中等重要 | ○Very integral /非常重要 | ○Extremely integral /极其重要 |
| --- | --- | --- | --- | --- |

29. HB2: I am comfortable incorporating AI tools into my daily tasks. / 我习惯将AI工具融入我的日常任务中。

[单选题]

| ○Not integral /不重要 | ○Somewhat integral /有点重要 | ○Moderately integral /中等重要 | ○Very integral /非常重要 | ○Extremely integral /极其重要 |
| --- | --- | --- | --- | --- |

30. HB3: I am open to trying new AI tools that can improve my efficiency. / 我愿意尝试新的AI工具来提高我的效率。

[单选题]

| ○Not integral /不重要 | ○Somewhat integral /有点重要 | ○Moderately integral /中等重要 | ○Very integral /非常重要 | ○Extremely integral /极其重要 |
| --- | --- | --- | --- | --- |

**Behavioral Intention (BI) / 行为意向 (BI)**

31. BI1: I intend to use DeepSeek AI for future tasks. / 我打算在未来的任务中使用DeepSeek AI。

[单选题]

| ○Not integral /不重要 | ○Somewhat integral /有点重要 | ○Moderately integral /中等重要 | ○Very integral /非常重要 | ○Extremely integral /极其重要 |
| --- | --- | --- | --- | --- |

32. BI2: I will likely recommend DeepSeek AI to others. / 我很可能会向其他人推荐DeepSeek AI。

[单选题]

| ○Not integral /不重要 | ○Somewhat integral /有点重要 | ○Moderately integral /中等重要 | ○Very integral /非常重要 | ○Extremely integral /极其重要 |
| --- | --- | --- | --- | --- |

33. BI3: I use DeepSeek AI regularly for my tasks. / 我定期使用DeepSeek AI完成任务。

[单选题]

| ○Not integral /不重要 | ○Somewhat integral /有点重要 | ○Moderately integral /中等重要 | ○Very integral /非常重要 | ○Extremely integral /极其重要 |
| --- | --- | --- | --- | --- |

**Usage Behavior (UB) / 使用行为 (UB)**

34. UB1: How often do you use DeepSeek AI for tasks? / 您多久使用一次DeepSeek AI完成任务？

[单选题]

| ○Never /从不 | ○Rarely /很少 | ○Monthly /每月 | ○Weekly /每周 | ○Daily /每天 |
| --- | --- | --- | --- | --- |

35. UB2: How satisfied are you with the overall experience of using DeepSeek AI? / 您对使用DeepSeek AI的整体体验有多满意？

[单选题]

| ○Less than a month /不到一个月 | ○1-6 months / 1-6个月 | ○6-12 months / 6-12个月 | ○12-24 months / 12-24个月 | ○More than 24 months /超过24个月 |
| --- | --- | --- | --- | --- |

36. UB3: When given the choice, how likely are you to choose DeepSeek AI over other AI tools? / 如果可以选择，您有多大可能选择DeepSeek AI而不是其他AI工具？

[单选题]

| ○Not integral /不重要 | ○Somewhat integral /有点重要 | ○Moderately integral /中等重要 | ○Very integral /非常重要 | ○Extremely integral /极其重要 |
| --- | --- | --- | --- | --- |

Additional Comments / 附加评论

37. Please share any additional comments or suggestions about using DeepSeek AI. / 请分享您对使用DeepSeek AI的其他意见或建议。

[填空题]

_________________________________
